# Self-rehabilitation strategy for rural community-dwelling stroke survivors in a lower-middle income country: a modified Delphi study

**DOI:** 10.1371/journal.pone.0303658

**Published:** 2025-02-25

**Authors:** Rabiu Ibrahim, Conran Joseph, Aimée Stewart, Isa Usman Lawal

**Affiliations:** 1 Directorate of Health Services, Department of Hospital Services, Physiotherapy Division, National Assembly Abuja, Nigeria; 2 Department of Health and Rehabilitation Sciences, Division of Physiotherapy, Stellenbosch University, Cape Town, South Africa; 3 Department of Physiotherapy, Faculty of Health Sciences, University of the Witwatersrand, Johannesburg, South Africa; 4 Department of Physiotherapy, Faculty of Allied Health Sciences, College of Health Sciences, Bayero University, Kano, Nigeria; Ministry of Health, Sri Lanka, SRI LANKA

## Abstract

**Background:**

More than half of stroke survivors in lower-middle income countries lack access to stroke rehabilitation services. The promotion of self-rehabilitation could be promising for addressing stroke rehabilitation inadequacies in lower-middle income countries. Self-rehabilitation interventions are more readily acceptable to community-dwelling stroke survivors, and therefore, have the potential to boost the successful realization of the Sustainable Development Goals and other WHO rehabilitation goals. We report a consensus-building process that sought to identify which task trainings are relevant to include in a task-specific self-rehabilitation strategy for rural community-dwelling stroke survivors.

**Methods:**

An iterative two-stage mixed-method consensus-building approach was used: (1) focus group discussions (n =  5) with rural community-dwelling chronic stroke survivors were conducted to explore personal life experiences in performing daily activities, and the results were used to develop a list of candidate task trainings that could be included in a task-specific self-rehabilitation intervention model for improving functional ability of survivors; (2) a three-round Delphi exercise with a panel of stroke rehabilitation experts to establish consensus on the importance/relevance of the developed task trainings. Consensus was pre-defined to be the point where the proportion of items given a rating of 3 (quite relevant) or 4 (highly relevant) by expert panellists is ≥  0.8. Kendall’s coefficient of concordance (W) was used to assess the level of agreement among the expert panellists.

**Results:**

A list of 74 task trainings was generated from the results of the focus group discussions involving 29 chronic stroke survivors. The tasks were classified as follows: training for the upper extremity (37); lower extremity training (21); trunk training (7); and balance training (9). A panel of 13 stroke rehabilitation experts reviewed these task trainings using the Delphi method and consensus was reached on keeping 28 task trainings in the first round (Kendall’s W =  0.252, p <  0.001) and an additional 7 in the second round (Kendall’s W =  0.409, p <  0.001). In the study team’s analysis of open text responses, several areas of debate were identified and some task trainings were modified. The exercise yielded 49 task trainings (66% of 74) on which there was consensus (the mean proportion of items given a rating of 3 or 4 by panellists was 0.93; Kendall’s W =  0.291, p <  0.001) to keep 3 task training groups relating to: upper extremity (27), lower extremity/balance (8), trunk strength (4) as well as warm up exercises (10).

**Conclusion:**

The study provides a consensus-based view of the features of a task-specific self-rehabilitation training strategy to improve outcomes following a stroke. This self-rehabilitation training strategy can be used as an intervention approach to augment and promote stroke rehabilitation among rural community-dwelling stroke survivors, especially in sub-Saharan Africa.

## Introduction

Stroke is a substantial source of acquired adult neurological disability, and its burden and associated risk factors have increased worldwide from 1990 to 2021 [1,2]. In low- and middle-income countries (LMICs), stroke incidence has increased alarmingly in the past decade [2,3]. The consequences of stroke have long-lasting effects and require long-term management of the ensuing limitations. Lack of resources, inadequate numbers of rehabilitation professionals, poor awareness, and lack of technical capacity have made the accessibility and availability of stroke rehabilitation services difficult in LMICs, particularly in the rural areas of sub-Saharan Africa (SSA) [4].

Currently, the need for stroke rehabilitation is largely unmet. In some regions of LMICs, more than 50% of people do not receive the rehabilitation services they require [5]. Disturbances, including conflicts, disasters and outbreaks of infectious diseases, create massive surges in rehabilitation needs while also disrupting any available rehabilitation services. The effect of the recent COVID-19 pandemic and its associated physical distancing protocols, and other emerging communicable diseases have added to the already existing challenges to stroke rehabilitation delivery in many regions of LMICs.

In Nigeria, like in other LMICs, there is no existing intervention strategy to support rehabilitation among rural community-dwelling stroke survivors. Rehabilitation professionals employ the practice of prescribing certain activities termed ‘home programme’ for stroke survivors to carry out at home. These activities are not always task-specific and none has been established through a proper expert consensus approach.

Self-rehabilitation that includes task-specific training could be promising for addressing stroke rehabilitation inadequacies in LMICs. Self-rehabilitation interventions have been shown to improve outcomes post-stroke, lessen the risk of stroke relapse and have encouraging impacts on healthcare resource utilization, which is of great significance in LMICs [6–8]. Moreover, having the advantage of being carried out in the comfort of the individual’s home, self-rehabilitation interventions can easily be practiced by many community-dwelling stroke survivors, and therefore, have the potential to promote wider stroke rehabilitation coverage and drive towards the successful realization of SDG 3 – “Ensuring healthy lives and promoting well-being for all at all ages” [9] and other WHO rehabilitation goals [10].

To develop a structured task-specific self-rehabilitation strategy for community-dwelling stroke survivors, the Delphi approach would be most appropriate to achieve relevant experts’ consensus on the task trainings to be included. Also, stroke survivors should be consulted in developing the initial task items of the model. In research generally, the Delphi method is used to build consensus around a particular research question or topic. Experts could be surveyed via questionnaires without being physically assembled in a modified Delphi technique [11]. The Delphi method is usually used in healthcare when guidelines or treatment protocols need to be developed and evidence is limited or inconsistent [12].

The Delphi method is particularly well-suited for developing a task-specific self-rehabilitation strategy due to several key advantages. Firstly, it facilitates the inclusion of experts from various disciplines related to stroke rehabilitation, ensuring a broad range of perspectives that enrich the development of the rehabilitation model. Additionally, by enabling anonymous responses, the Delphi method minimizes the influence of dominant voices and encourages honest feedback, fostering open exchanges that are crucial for addressing complex rehabilitation needs. Finally, its iterative nature allows for the refinement of ideas through multiple rounds of questioning, enabling experts to reconsider their opinions based on group feedback, ultimately leading to a stronger consensus on the most relevant task-specific trainings.

The primary objective of this study was to build expert consensus on the appropriate items (task trainings) to be included in a task-specific self-rehabilitation training strategy that could be administered among Hausa-speaking community-dwelling stroke survivors in Nigeria.

## Methods

Ethical approval for this study was sought and obtained from the College Health Research Ethics Committee of Bayero University, Kano (ref: NHREC/06/12/5). The ACCORD (Accurate Consensus Reporting Document) guideline [13] was followed in reporting this study.

A two-stage consensus-building approach was adopted in this study. The first stage was the identification of relevant tasks that could be included, based on importance, in a task-specific self-rehabilitation intervention model for improving functional ability among community-dwelling stroke survivors in a resource-limited setting (September-December 2022). The second stage comprised the conduct of a modified Delphi exercise to reach a consensus on the final list of task trainings to include in the intervention model (January 2023-January 2024).

### Stage 1: Identifying the task training items to include in the intervention model

A list of potential task trainings that could be included in the intervention model was drawn from information gathered through focus group discussions with stroke survivors. Focus group discussion was similarly used in a previous study to obtain the initial content of a Delphi study instrument [14].

#### Focus group discussions (FGDs).

Five FGDs involving twenty-nine community-dwelling chronic stroke survivors were conducted (between September and December 2022) in Kura town of Northern Nigeria, with the aim of identifying the common daily activities that stroke survivors find difficult to accomplish. Kura town is located about 27km from Kano city and its inhabitants are predominantly Hausa farmers engaged in rice farming.

#### FGD: participants and sampling strategy.

In order to generate diverse information with a range of experiences in performing daily activities after stroke, participants were purposefully selected based on gender blend, age, and from diverse locations within the community. Local community contacts and some identified stroke survivors within the community helped with participant recruitment. All stroke survivors who expressed interest and met the following criteria were recruited into the study: aged 18 and above; diagnosed with ischemic or haemorrhagic stroke; stroke onset of more than six months; speak and understand the Hausa language; capable of giving informed consent.

Participants were excluded if they had: cerebrovascular events due to malignancy or head trauma; limited comprehension (receptive and/or expressive aphasia) as indicated by having a score of < 80 on the Comprehensive Aphasia Test [15]; been diagnosed with any other neurological/mental disorder.

#### FGDs: procedure.

FGD sessions were scheduled with those who gave consent and met the inclusion criteria. Four of the focus groups had 6 participants while one focus group had 5 participants. The discussions took place in a convenient classroom in one of the schools within Kura town. Using a semi-structured interview guide (S1) which was developed by two members of the research team (IUL and RI), the participants’ experiences in performing daily activities after stroke were explored.

Discussions were conducted in Hausa language and audio-recorded. Maximum duration of the FGDs was 1h 40 min. The FGDs were facilitated by IUL and field notes were taken by RI. The concepts of data saturation and collection of rich and thick data [16] were used to end the data collection.

#### Developing the initial task training items.

This was done in two steps; first, the results of the focus group discussions were considered by three physiotherapists (who were part of the research team) with more than 15 years’ experience in stroke rehabilitation. All the tasks that the stroke survivors reported as being difficult were identified. Secondly, the three physiotherapists in collaboration with a clinical kinesiologist (who obtained Master’s degree in kinesiology, also, having more than 15 years working experience in a rehabilitation centre) isolated and grouped similar tasks based on the pattern of movement and the body part (i.e. upper extremities, lower extremities, and the trunk) involved. The training requirements in each group were further categorized based on the activities. This process resulted in the generation of a list of potential tasks (S2) to include in the intervention model which was used in the Delphi exercise.

### Stage 2: Consensus-building to select and refine the tasks to include in the intervention model

We used a modified Delphi technique [17] to build consensus on which tasks generated from stage 1 were relevant to include in the intervention model. Three rounds of rating and review by an expert panel were conducted over an eleven-month period.

#### Selection of panellists.

Using a mixture of purposive and stratified sampling techniques, 30 stroke rehabilitation experts (comprising 10 from Nigeria, 10 from other parts of Africa and 10 from other parts of the world) from diverse professional groups involved in stroke rehabilitation, with at least 15 years of experience in stroke management, were contacted through email for their consent (S3) to participate in the Delphi process. The selection criteria considered for the Delphi panellists included healthcare professionals such as physiotherapists, occupational therapists, neurologists, and rehabilitation specialists who work directly with stroke survivors, as well as researchers who have published studies on stroke rehabilitation or self-rehabilitation strategies. We emphasized the importance of experts with hands-on experience in stroke rehabilitation, particularly those focusing on task-specific strategies, and researchers who have made significant contributions to the field. Additionally, geographical diversity was prioritized to ensure representation from various regions, accounting for cultural and systemic variations in rehabilitation practices. Finally, it was essential that experts were currently active in their fields, as this increases the likelihood that they remain informed about the latest research and practices.

Potential panellists were identified through their published work in the field of stroke rehabilitation and some were suggested by the main study team. These potential panellists were also asked to suggest others who they considered suitable to participate (snowball sampling), and these were also invited, provided they met the inclusion criteria. Our aim was to achieve a panel of 11 to 30 panellists, which is a range of sample size considered effective and reliable for the Delphi technique [18,19].

To determine the expertise of potential panellists, we reviewed their academic publications, presentations, and involvement in research projects related to stroke rehabilitation. We also sought recommendations from trusted colleagues or institutions to identify individuals recognized for their expertise in this area. In cases where a potential panellist’s expertise was difficult to ascertain through these methods, we directly inquired via email about their personal experience, views on rehabilitation, and familiarity with task-specific strategies.

To minimize potential selection bias, a broad selection criterion was employed to include a diverse array of experts from various disciplines, backgrounds, and geographical locations. A stratified sampling technique based on geographical areas (countries and regions) was also utilized to ensure representation from different perspectives. Panellists were asked to disclose any potential conflicts of interest that could influence their views, enabling us to consider excluding individuals with significant biases. Furthermore, we ensured that confidentiality and anonymity were maintained throughout the Delphi process to encourage honest feedback and minimize the influence of dominant personalities. A copy of the set of tasks, including an instruction note (S4) on how to rate the items, was sent to each of those who met the inclusion criteria and gave their consent to participate. All correspondence with the panellists was done via e-mail.

#### Defining consensus.

Consensus on a topic or an issue can be regarded as the general agreement among a group of people that are well informed (experts) about the topic or issue in question. In the literature, consensus was defined as the “gathering of individual evaluations around a median response with minimal divergence” [20,21]. While experts can reach 100% agreement on only a few issues [22], consensuses are determined by a certain percentage of experts who agree to an issue in a Delphi survey, and this should be defined and stated before the conduct of the survey [23].

In this study, the level of consensus regarding the relevance of task training was measured at the conclusion of both the first and second rounds of assessment. The final level of consensus on the overall suitability of the intervention model was evaluated at the end of the third round. To quantify the consensus on the relevance of each specific task training, we employed a systematic approach. For each task, we calculated the proportion of experts who rated it as 3 or 4 on a Likert scale, where 1 represented “not relevant” and 4 signified “highly relevant.” This calculation involved dividing the number of experts who rated a task training as 3 or higher by the total number of experts participating in the evaluation.

A consensus was deemed achieved if a task training received a score of 0.78 or higher [24], indicating that a significant majority of the panellists recognized its relevance. Conversely, any task training that scored below this threshold ( < 0.78) was either modified for further consideration in the subsequent round or removed from the evaluation process entirely, ensuring that only the most pertinent tasks were advanced.

For assessing the overall suitability of the intervention model, we determined the level of consensus through a similar methodology. Specifically, we computed the proportion of items rated as 3 or 4 by the panellists involved, following the guidelines established by Waltz and Bausell [25]. A score of 0.8 or above was accepted as indicative of moderate to high consensus, based on existing procedural evidence from the literature [22,26–28]. This threshold helped to ensure that the intervention model reflects a broad agreement among experts, ultimately enhancing the reliability and applicability of our findings in real-world rehabilitation settings.

#### Delphi round 1.

In this round, the panellists rated each item of the model in terms of its relevance to the underlying construct. The item ratings were on a 4-point scale to avoid a neutral and ambivalent midpoint [24] and to produce stable findings in Delphi studies [19]. The rating interpretation was as follows: 1 =  not relevant, 2 =  somewhat relevant, 3 =  quite relevant and 4 =  highly relevant. After all the panellists had finished their ratings, the package was retrieved and computation to determine consensus on each item’s relevance was done. Items that were not rated by at least two-thirds of the panellists were not included in the computation. These were regarded as items on which no consensus was reached and were included in the next round.

For each task item, options for the panellists to provide text comments in support of their rating, reason for abstaining from rating or to suggest changes to the task were available. The panellists were given a period of three weeks to respond, and reminders were sent on a weekly basis.

#### Delphi round 2.

In this round, the written opinions of all the panellists were summarized and shared among them. Experts were invited to consider their scores for the remaining task trainings that did not make the required item validity score and were not removed, based on group responses in relation to the overall responses received. They suggested what they deemed appropriate between their rating and the average opinion of other respondents. Additionally, further explanation of some culture-based tasks was made available to the panellists, particularly, those who were not familiar with the culture of the intervention target population. Panellists were also asked to give written opinions (S5) on the overall structure and inclusiveness of the included tasks. The item rating and validity score computation was as in round 1.

Prior to the commencement of the third round, an in-person meeting of the study team was held, purposely to decide on some tasks which are peculiar to the culture of the study area and that some panellists were not familiar with, and as a result had abstained from rating. The study team also finalized the structure and inclusiveness of the task training items in the model by considering the comments from the panellists in round 2.

The research team members who are Hausa natives played two major roles. First, they served as cultural brokers by facilitating understanding between Western rehabilitation concepts and Hausa cultural context, and second, they provided insight into Hausa cultural values, customs and healthcare practices, and clarified some cultural issues. It was ensured that all decisions taken during the research team meeting were strictly based on the recommendations and responses from the panellists. All items that had the required score and did not have suggestions made about them by the panellists, were not debated in the meeting and panel consensus was deemed final.

#### Delphi round 3.

Round 3 involved content validation of the task training items in the model (including those that had been retained in rounds 1 and 2, and those modified or added during the in-person study team meeting. In this round, panellists were invited to reassess the content and rate the whole model using the criteria in round 1. All responses were collected individually.

#### Data analysis.

FGD**:** The audio recordings of the focus group discussions were transcribed verbatim and anonymized with codes. The Hausa language transcript was translated into English at the English Language department of Bayero University, Kano. Using the deductive open coding process, two members of the research team (IUL and RI) agreed on an initial flat coding frame, developed a set of predetermined codes based on the study’s objective, both read through the data line by line, and assigned excerpts to codes until themes were developed.

Delphi: Analysis of data was done using Microsoft Excel and IBM SPSS Statistics 23. Analyses involved calculating the proportions of agreement and Kendall’s coefficient of concordance (W). For the first and second rounds of the Delphi, for each task training, the number of experts who gave a rating of 3 or 4 was computed and divided by the total number of experts. A score of ≥  0.78 indicated that consensus had been reached to keep the particular task training [24].

In the third round of the Delphi study, the number of items given a score of 3 or 4, divided by the total number of items was calculated for each panellist (following the methodology outlined by Waltz and Bausell) [25], then the results were averaged and considered. A threshold of ≥  0.8 was deemed a consensus [26–28]. In each round, Kendall’s W was used to assess the level of agreement and statistical significance.

## Results

### The FGD

Five focus group discussions were conducted, with an average duration of 76 minutes (range =  55–96 minutes) each and five to six participants per group. A total of 29 chronic stroke survivors (median stroke onset =  31 months) living in the community, with mean age of 52.14 ±  10.144 years, were included in the study. The majority of the participants (59%) were over the age of 50. Two-thirds of the respondents were men (65.5%), and more than three-quarters were married (82.8%). Almost half (48.3%) of them had no formal education, and about 40% were skilled agricultural, forestry, and fishery workers (based on the International Labor Organization classification of occupation) [29]. The socio-demographic and clinical characteristics of the FGD participants are presented in Table 1.

**Table 1:** Characteristics of the FGD participants.

Variable	n	%
**Gender**		
Male	19	65.5
Female	10	34.5
**Level of Education**		
Informal	14	48.3
Primary	6	20.7
Secondary	5	17.2
Tertiary	4	13.8
**Marital status**		
Single	2	6.9
Married	24	82.8
Divorced	1	3.4
Widow/widower	2	6.9
**Occupation**		
Technicians and associate professionals	3	10.3
Service and sales workers	6	20.7
Skilled agricultural, forestry, and fishery workers	11	37.9
Craft and related trade workers	2	6.9
Elementary occupation	1	3.4
None	6	20.7
**Hemiparesis side**		
Right	13	44.8
Left	16	55.2
**Arm dominancy**		
Right	23	79.3
Left	6	20.7

Three major themes and eight sub-themes were identified (Table 2). The themes were related to the most commonly reported activities/tasks which stroke survivors struggled to perform and included personal care activities, religious activities, and daily participation activities.

**Table 2:** Themes and sub-themes.

Themes	Sub-themes
Personal care	Feeding
	Dressing/undressing
	Bathing
	Washing
Religious activities	Ablution
	Praying
Participation	Standing/walking
	Cooking

The themes are described below with some examples of anonymized quotes.

#### Personal care.

The major activities reported under this theme are categorized into feeding, dressing/undressing and bathing and washing. Many mention the difficulty of performing basic tasks, such as eating, dressing, bathing, and washing clothes, using only their unaffected side. For instance, one person has to eat with their left hand, despite disliking it, while another struggles to tie a wrapper and requires assistance from a family member. Several participants mention relying on a walking stick for mobility, needing help from loved ones for dressing and bathing, or performing tasks like washing clothes on their own.“… I have no option but to eat with the left hand... I don’t like it, but I have to do it.” (Female, 50 yrs, right-sided hemiparesis)

“It is always difficult to tie a wrapper around my waist, because I use one hand, so my daughter has to assist me in tying it.” (Female, 38 yrs, right-sided hemiparesis)

#### Religious activities.

The participants were predominantly Muslims and many of them reported difficulty in performing ablution (sequential washing of some body parts which is a pre-requisite for performing the Islamic daily prayers) and praying:

“In the aspect of performing religious rituals, I am weak; thank God that now I can bend down during prayers, but I can’t prostrate. Also I perform ablution slowly, the person that assists me is also not feeling well now. So somehow, I manage to do it, even though my hand is heavy.” (Male, 51 yrs, left-sided hemiparesis)

#### Social participation activities.

The participants describe various mobility challenges due to their hemiparesis, highlighting the limitations they face in daily activities. One person recounts falling off a motorcycle after attempting to use their weaker left leg, which led to frustration.

“… For example, when I want to stand up to walk, I do that from the right side of the body because it is the stronger side. Just not long ago, I fell down while trying to get off a motorcycle. I tried getting down from the left side and placed the left leg on the ground first, but it couldn’t carry me and gave way, so I fell down... it was frustrating.” (Male, 48 yrs, left-sided hemiparesis).

Another participant struggles with climbing, as stepping up with the affected leg causes them to become stuck, though stepping with the unaffected leg first helps. A female respondent discusses how her condition has made it impossible to continue her work, such as frying and selling groundnuts, leading to her dependence on others for basic needs.

“… You go out every day to work and earn a living for you and your family, but now I can’t. I rely totally on others to give me … I used to fry groundnuts and sell, but I can’t do that now. Because of the sickness, I am not able to cook.” (Female, 50 yrs, right-sided hemiparesis)

### The Delphi

Seventy-four (74) task training items (S2) were drawn and designed as potential items for the stroke self-rehabilitation model, based on information gathered through the focus group discussions with stroke survivors (Table 3).

**Table 3:** Initial grouping/categorization and number of the task training items.

Task training group	Category	No of task training items
**Upper extremity training**	Trainings for reaching	13
	Trainings for grasp/grip	4
	Trainings for moving objects	6
	Trainings for object manipulation	10
	Trainings for hand/fingers precision	4
**Group total**		**37**
**Lower extremity training**	Training for transfers from sit to stand	9
	Training for maintaining standing	4
	Training for reaching in standing	3
	Training for stepping and walking	5
**Group total**		**21**
**Trunk training**	Training for trunk strength in sitting	4
	Training for trunk strength in lying position	3
**Group total**		**7**
**Training for balance**	Training for balance in sitting	3
	Training for balance in standing	6
**Group total**		**9**
**Total task training items**		**74**

### Consensus-building to select and refine the tasks to include in the intervention model

Forty-five experts who met the selection criteria were invited. Eighteen panellists consented to participate, of whom 13 (72%) completed the 3 rounds of the Delphi study (Table 4). The Delphi technique took almost a year (January 2023-January 2024) to complete due to delays in response from the panellists.

**Table 4:** Demographic characteristics of the panellists.

Panellists (n = 13)	
**Gender**	
Male	6 (46.2%)
Female	7 (53.8%)
**Country of residence**	
Nigeria	7 (53.8%)
South Africa	4 (30.8%)
Iran	1 (7.7%)
Thailand	1 (7.7%)
**Experience in stroke rehabilitation**	
Mean number of years	22 years

At the end of Delphi round one (S6), 28 (38%) of the task training items for the intervention model achieved the threshold of ≥  0.78, with good agreement among the panellists (Kendall’s W =  0.252, p <  0.001), and so were retained. Four of the items received scores of ≤  2 by all the panellists and these items were removed. The remaining 42 items were amended based on the comments and suggestions by the panellists (S5) and re-entered into round two (S7). At the end of the second round, 7 (17%) of the items achieved the threshold of ≥  0.78 and were retained.

**Fig 1:**
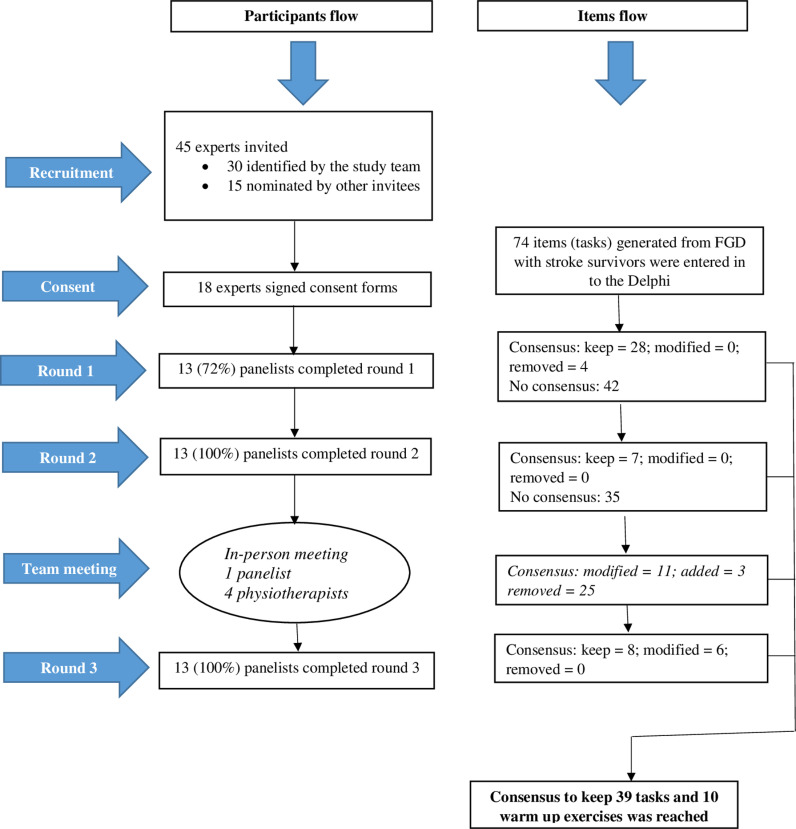
Flow of the consensus-building process.

Thus, at the end of the second round, 35 (47%) of the 74 task trainings met the predefined threshold of ≥  0.78 while 4 (5%) did not and were removed. The consistency of the rating was good (Kendall’s W =  0.409, p <  0.001). The remaining 35 items were considered at the study team meeting. The research team (four physiotherapists and one Delphi panellist, all Hausa natives) agreed on: the removal of all 25 items proposed as meriting removal; the 10 items modified as warm up exercises; and the 4 new task trainings that were added, based on the comments and suggestions (S5) made by the panellists in both previous rounds of the Delphi. Fourteen training tasks were thus added to the already retained 35 items and entered into round three (S8). Fig 1 shows the flow of participants and items through the stages of the study.

At the end of round three, an average threshold score of 0.93 (Table 5) was achieved and there was good agreement among the panellists (Kendall’s W =  0.291). This agreement was statistically significant (p <  0.001), therefore all the items were retained.

**Table 5:** The proportion of items given a rating of 3 or 4 by panellists involved in round 3.

Panellist	Score
P1	0.79
P2	1.00
P3	0.92
P4	0.95
P5	0.89
P6	0.95
P7	0.97
P8	0.97
P9	0.97
P10	0.97
P11	0.82
P12	0.92
P13	1.00
**Mean score**	**0.93**

The number of items in each category through the stages of the study is shown in Table 6. Thus at the end of round three, consensus was reached to retain 49 (66%) task trainings (Table 7) of which 10 were retained as warm up exercises (S9).

**Table 6:** Number (%) of items in each category through the stages of the study.

Stage 1		Stage 2										
Group	Category	Round 1		Round 2		Study team meeting				Round 3		
		No. of items	Items to keep	No. of items	Items to keep	No. of items	Items removed	Items modified	Items added	No. of items	Items to keep	Final no. of items in each category
Upper extremity training	Reaching in sitting	13	4 (31%)	5	0 (0%)	5	5	0	0	4	4	4
	Grasp/grip*	4	2 (50%)	2	0 (0%)	2	1	1	0	3	3	3
	Moving objects*	6	3 (50%)	3	0 (0%)	3	1	2	0	5	5	5
	Objects manipulation	10	4 (40%)	6	3 (50%)	3	0	3	0	10	10	10
	Hand/fingers precision	4	4 (100%)	0	0 (0%)	0	0		1	5	5	5
Lower extremity training^¥^	Sit to stand	9	2 (22%)	7	0 (0%)	7	4	4	0	6	6	6
	Maintaining standing^ ∞ ^	4	1 (25%)	3	0 (0%)	3	3	0	0	1	1	1
	Reaching in standing^ ∞ ^	3	1 (33%)	2	0 (0%)	2	2	0	0	1	1	1
	Stepping and walking	5	3 (60%)	2	1 (50%)	1	1	0	0	4	4	4
Training for trunk	Trunk strength in sitting^¶^	4	3 (75%)	1	0 (0%)	1	1	0	2	5	5	5
	Trunk strength in lying^¶^	3	1 (33%)	2	0 (0%)	2	2	0	0	1	1	1
Balance training^¥^	Maintaining balance in sitting	3	0 (0%)	3	1 (33%)	2	1	0	1	2	2	2
	Maintaining balance in standing	6	0 (0%)	6	2 (33%)	4	4	0	0	2	2	2
Total		74	28 (38%)	42	7 (17%)	35	25	10	4	49	49 (100%)	49 (66% of 74)

Note:

* grasp/grip and moving objects categories were merged; ^∞ ^ maintaining and reaching in standing were merged; ^¥^ lower extremity and balance training groups were merged; ^¶^ trainings for trunk strength in sitting and in lying were merged into one category.

**Table 7:** Final grouping/categorization of items in the model.

Task training group	Category	No of task training
**Upper extremity training**	Warm up	5
	Training for reaching	4
	Training for grasp/grip and Training for moving objects	8
	Training for object manipulation	10
	Training for hand/fingers precision	5
**Group total**		**32**
**Lower extremity and balance training**	Warm up	2
	Training for transfers from sit to stand	2
	Training for maintaining standing and reaching in standing	2
	Training for stepping and walking	4
**Group total**		**10**
**Training for trunk strength**	Warm up	3
	Training for trunk strength in sitting and lying position	4
**Group total**		**7**
**Total task training**		**49**

### Task-specificity of the items

The text responses of the panellists emphasized the importance of making the task training items in the model task-specific. As a result, items that comprised activities that were not task-specific received low ratings. The need for warm-up exercises prior to the commencement of the main training was also among the suggestions made by the panellists. In response to these comments and suggestions, the study team decided to retain some items comprising non-task-specific activities as warm-up exercises, and this decision was unanimously agreed by the panellists in the final round of the Delphi. Ten of the items in the model were converted to warm-up exercises: five for the upper extremity training group; two for the lower extremity and balance training group; and three for the trunk training group.

### Redundant and similar items

The panel highlighted the issue of repetition of items within and outside the categories. Redundancy and similarities were the major terms used by the panellists when describing such items; thus it was suggested that some items be merged. The panellists agreed that most of the items in the lower extremity training group were similar to those in the balance training group and could be used to improve both lower extremity and balance functions, consequently the lower extremity and balance training groups were merged into one training group. The following categories were merged during the study team meeting based on the panellists’ suggestions: grasp/grip and moving objects; maintaining and reaching in standing; training for trunk strength in sitting and in lying.

### Non familiar items

Some items involved activities that are peculiar to the culture of the target population and therefore were not clear to some panellists, which prompted them to suggest that, “use of pictures for these activities would help because it takes time to figure out what is expected.” Thus, picture demonstrations of such activities involving the use of Hausa local cap, eating bowl, and ablution-related activities were included in the second round of the Delphi. This resulted in better understanding and facilitated the rating of the items by the panellists.

## Discussion

This article presents a consensus-building study aimed at identifying relevant and suitable items (task trainings) for a Task-specific Self-Rehabilitation Training (TASSRET) intervention model, designed to improve functional ability outcomes among rural community-dwelling stroke survivors. The outcome is a list of 39 task-specific training exercises and 10 warm-up exercises that can be self-administered at home, utilizing common household items relevant to the target population.

In developing countries, health care systems often face limited economic resources, which restricts access to new and expensive technologies typically available in developed countries [4]. The introduction of innovative and cost-effective stroke intervention approaches would significantly enhance stroke rehabilitation efforts, particularly in these settings [30]. A previous study [8] demonstrated the feasibility of a self-rehabilitation programme for stroke survivors in a resource-limited context yielding encouraging results. However, the components of that intervention were not derived from expert consensus, and stroke survivors were not involved in the programme’s development, raising potential validity concerns.

Recent systematic reviews have identified several self-rehabilitation [7] and self-management [31] strategies and documented the equality in efficacy for post-stroke functional mobility outcomes with conventional therapy.; however, these strategies were predominantly developed and implemented in high-income countries (HICs) and may not be applicable to the low-resource settings of low- and middle-income countries (LMICs). Research by Hughes et al. [32] has indicated that rehabilitation experts from the US and Ethiopia have differing opinions on the importance and relevance of features to include in stroke tele-rehabilitation programmes. This disparity likely arises from cultural and resource differences, highlighting the necessity for rehabilitation programmes to be tailored to the geographical and cultural context of end users as demonstrated by the current study.

The TASSRET model is the product of collaborative input from rural community-dwelling stroke survivors and insights from 13 stroke rehabilitation professionals with extensive clinical experience. The findings of this study may enhance the currently inadequate stroke rehabilitation services and promote rehabilitation efforts in rural communities, particularly in sub-Saharan Africa. Rehabilitation is a critical component of universal health coverage and a key strategy for achieving Sustainable Development Goal 3: “Ensure healthy lives and promote well-being for all at all ages” [9]. The TASSRET model may foster inclusivity and expand access to rehabilitation services, enabling self-rehabilitation for rural community-dwelling stroke survivors — including those who are illiterate — within the comfort of their homes.

Two versions of the TASSRET model will be produced for implementation among community-dwelling stroke survivors: 1) a video version in the form of an Android mobile application, which demonstrates task training with visual and auditory cues, and 2) a booklet version, which includes written instructions, images, and descriptions of the task trainings.

This study has some strengths and limitations. The initial list of task trainings for entry into the first Delphi round was generated through focus groups with stroke survivors. This is recommended as a valid source of appropriate items to inform the first quantitative round of a Delphi survey [33]. Several rounds of written detailed information were sent to panellists to ensure that the panellists have proper understanding of the study’s aim and processes, and this helped to build good research relationships between the researchers and the panellists [34]. Moreover, reminder emails and sometimes phone calls were employed to enhance time and rate of response. Nonetheless, the response was at times poor.

A further strength of this study was the administrative skills employed in the coding system for tracking panellists and their responses through the Delphi rounds. Analysing changes of opinion and suggestions was undertaken and managed well. These are issues upon which the smooth execution of a Delphi is based, but which are scarcely considered in the literature [33].

One significant limitation of this Delphi study lies in the modification of certain task trainings prior to the final round of consensus building. Although these modifications were informed by the responses of the panellists, they diverge from the fundamental principles of the Delphi method, which typically emphasizes a structured process with minimal intervention from the research team. The involvement of the study team in item modification was deemed necessary to facilitate progress, particularly in light of the complexities encountered during the study. This necessity aligns with findings reported by Green et al. [35] in their Delphi study examining the information requirements of general practitioners, where the research team similarly reordered and condensed the generated information to enhance clarity and relevance.

The decision to adapt the items was largely influenced by the distinct cultural and contextual factors pertinent to the target population — Hausa-speaking community-dwelling stroke survivors in Nigeria. By integrating local insights and experiences, the research team aimed to ensure that the final model would be more effectively aligned with the specific needs of this demographic. The cultural nuances inherent in the Hausa context are critical for the development of a model that truly resonates with the lived experiences of the participants.

Efforts were made to ensure that the study procedures adhered closely to the original Delphi methodology, particularly concerning all major task training items included in this research. Modifications were implemented only for items that required cultural or contextual adjustments. We believe that these adaptations do not compromise the quality of our findings.

Another notable limitation of this Delphi study pertains to the challenge of achieving true anonymity among the panellists. To maximize response rates and ensure comprehensive participation, the research team retained knowledge of the identities of certain panellists. This familiarity proved instrumental in encouraging late respondents to contribute their insights. However, it is important to note that while the research team was aware of the panellists’ identities, every effort was made to ensure that individual panellist responses remained strictly confidential from one another. This measure was intended to foster an environment of open and honest dialogue, although it may have inadvertently impacted the perceived anonymity of the process.

Additionally, the process of identifying potential panellists posed challenges that could affect the study’s generalizability. The contact information for many of the potential panellists was sourced from their published academic papers, which, in some cases, contained outdated addresses or affiliations. This not only complicated the recruitment process but also may have limited the diversity of perspectives represented in the panel. Future research endeavours may benefit from employing a more comprehensive strategy for identifying and recruiting panellists, potentially utilizing multiple databases or networks to enhance the accuracy and currency of contact information. Also, due to limited resources and time, we did not include the family caregivers in the FGD, which we feel, if we did, would have added value to the initial task-training items. We therefore recommend future studies to explore the opinions and perceptions of stroke survivors’ caregivers regarding the intervention model.

These limitations underscore the importance of balancing methodological rigour with practical considerations in the Delphi process. While efforts were made to maintain confidentiality and encourage participation, the implications of panellist identification and recruitment challenges warrant careful consideration in interpreting the findings of this study.

## Conclusion

This study successfully established a consensus on a list of 39 task-specific training activities and 10 warm-up exercises that can be integrated into a self-rehabilitation strategy for stroke survivors. The proposed Task-Specific Self-Rehabilitation Training model presents a viable intervention to enhance and support stroke rehabilitation among rural community-dwelling stroke survivors, particularly in sub-Saharan Africa.

Further investigations are warranted to assess the effectiveness of this intervention programme in improving functional outcomes for stroke survivors, ultimately aiming to enhance their physical functioning and quality of life post-stroke.

## Supporting information

S1 File FGD interview guide. (DOCX)

S2 File Initial task trainings. (DOCX)

S3 File Delphi invitation and consent letter. (DOCX)

S4 File Delphi instruction note. (DOCX)

S5 File Panellists’ comments and suggestions. (DOCX)

S6 File Delphi round 1. (ZIP)

S7 File Delphi round 2. (ZIP)

S8 File Delphi round 3. (ZIP)

S9 File Final task trainings (TASSRET). (DOCX)

S10 File SPSS analyses output. (DOC)

## References

[1] Langhorne P, Bernhardt J, Kwakkel G. Stroke rehabilitation. Lancet. 2011;377(9778):1693–702. doi: 10.1016/S0140-6736(11)60325-5 21571152

[2] CegolonL; GBD 2021 Stroke Risk Factor Collaborators. Global, regional, and national burden of stroke and its risk factors, 1990–2021: a systematic analysis for the Global Burden of Disease Study 2021. Lancet Neurol. 2024;23(10):973–1003. doi: 10.1016/S1474-4422(24)00369-7 39304265

[3] Johnson W, Onuma O, Owolabi M, Sachdev S. Stroke: a global response is needed. Bull World Health Organ. 2016;94(9):634–A. doi: 10.2471/BLT.16.181636 27708464 PMC5034645

[4] Tawa N, Rhoda A, Brink Y, Urimubenshi G, Giljam-Enright M, Charumbira MY, et al. Stroke rehabilitation services in Africa – Challenges and opportunities: A scoping review of the literature. In: Louw Q, editor. Collaborative capacity development to complement stroke rehabilitation in Africa [Internet]. Cape Town (ZA): AOSIS; 2020. Chapter 1. Available from: https://www.ncbi.nlm.nih.gov/books/NBK574231/34606199

[5] Neill R, Shawar YR, Ashraf L, Das P, Champagne SN, Kautsar H, et al. Prioritizing rehabilitation in low- and middle-income country national health systems: a qualitative thematic synthesis and development of a policy framework. Int J Equity Health. 2023;22(1):91. doi: 10.1186/s12939-023-01896-5 37198596 PMC10189207

[6] Yan LL, Li C, Chen J, Miranda JJ, Luo R, Bettger J, et al. Prevention, management, and rehabilitation of stroke in low- and middle-income countries. eNeurologicalSci. 2016;2:21–30. doi: 10.1016/j.ensci.2016.02.011 29473058 PMC5818135

[7] Everard G, Luc A, Doumas I, Ajana K, Stoquart G, Edwards MG, et al. Self-rehabilitation for post-stroke motor function and activity-a systematic review and meta-analysis. Neurorehabil Neural Repair. 2021;35(12):1043–58. doi: 10.1177/15459683211048773 34696645

[8] Niama Natta DD, Lejeune T, Detrembleur C, Yarou B, Sogbossi ES, Alagnidé E, et al. Effectiveness of a self-rehabilitation program to improve upper-extremity function after stroke in developing countries: A randomized controlled trial. Ann Phys Rehabil Med. 2021 Jan;64(1):101413. Epub 2020 Oct 15. doi: 10.1016/j.rehab.2020.03.017 32619630

[9] UN General Assembly. Transforming our world: the 2030 Agenda for Sustainable Development; 21 October 2015, A/RES/70/1, [cited 6 August 2023]. https://www.refworld.org/docid/57b6e3e44.html

[10] WHO. Rehabilitation goals; 2030 [cited 6 August 2023]. https://www.who.int/initiatives/rehabilitation-2030

[11] de Villiers MR, de Villiers PJT, Kent AP. The Delphi technique in health sciences education research. Med Teach. 2005 Nov;27(7):639–43. doi: 10.1080/13611260500069947 16332558

[12] The Center for Health Design, 2017. Taylor, Joseph, Quan, & Nanda; 2014.

[13] Gattrell WT, Logullo P, van Zuuren EJ, Price A, Hughes EL, Blazey P, et al. ACCORD (ACcurate COnsensus Reporting Document): A reporting guideline for consensus methods in biomedicine developed via a modified Delphi. PLoS Med. 2024 Jan 23;21(1):e1004326. doi: 10.1371/journal.pmed.1004326 ; PMCID: PMC10805282 38261576 PMC10805282

[14] Steward IP, Young ES, Dogra SA, Stamp E, Daly-Smith A, Siddique K, et al; JU:MP research & development team. How to develop young physical activity leaders? A Delphi study. PLoS One. 2023 Sep 29;18(9):e0286920. doi: 10.1371/journal.pone.0286920 ; PMCID: PMC10540972. 37773961 PMC10540972

[15] Swinburn K, Porter G, Howard D. Comprehensive Aphasia Test (CAT). [Database record]. APA PsycTests; 2004. doi: 10.1037/t13733-000

[16] LeCompte MD, Schensul JJ. Designing and conducting ethnographic research: an introduction. Lanham, MD: AltaMira Press; 2010.

[17] Jones J, Hunter D. Consensus methods for medical and health services research. BMJ. 1995;311(7001):376–80. doi: 10.1136/bmj.311.7001.376 7640549 PMC2550437

[18] Dalkey NC. The Delphi method: an experimental study of group opinion. Santa Monica, CA: RAND Corporation; 1969. https://www.rand.org/pubs/research_memoranda/RM5888.html

[19] Akins RB, Tolson H, Cole BR. Stability of response characteristics of a Delphi panel: application of bootstrap data expansion. BMC Med Res Methodol. 2005;5:37. Published 2005 Dec 1. doi: 10.1186/1471-2288-5-37 16321161 PMC1318466

[20] Keenyey S, Hasson F, McKenna H. The Delphi Technique in Nursing and Health Research. A John Wiley & Sons, Ltd., Publication; 2011. p. 1–210.

[21] Ross S, Metcalf A, Bulger SM, Housner LD. Modified Delphi investigation of motor development and learning in physical education teacher education. Res Q Exerc Sport [Internet]. 2014;85(3):316–29. doi: 10.1080/02701367.2014.930087 25141085

[22] Mubarak N, Hatah E, Aris MAM, Shafie AA, Zin CS. Consensus among healthcare stakeholders on a collaborative medication therapy management model for chronic diseases in Malaysia; A Delphi study. PLoS One. 2019 May 10;14(5):e0216563. doi: 10.1371/journal.pone.0216563 ; PMCID: PMC6510413 31075110 PMC6510413

[23] Li Y, Ehiri J, Hu D, Zhang Y, Wang Q, Zhang S, et al. Framework of behavioral indicators for outcome evaluation of TB health promotion: a Delphi study of TB suspects and Tb patients. BMC Infect Dis. 2014;14(1):1–14. doi: 10.1186/1471-2334-14-268 24884569 PMC4030006

[24] Lynn MR. Determination and quantification of content validity. Nurs Res. 1986;35(6):382–5. doi: 10.1097/00006199-198611000-00017 3640358

[25] Waltz CF, Bausell RB. Nursing research; Design, statistics, and computer analysis. Philadelphia: F. A. Davis; 1981.

[26] Davis LL. Instrument review: Getting the most from a panel of experts. Applied Nursing Research. 1992 Nov;5(4):194–7. doi: 10.1016/s0897-1897(05)80008-4

[27] Grant JS, Davis LL. Selection and use of content experts for instrument development. Res Nurs Health. 1997 Jun;20(3):269–74. doi: 10.1002/(sici)1098-240x(199706)20:3<269::aid-nur9>3.0.co;2-g 9179180

[28] Polit D, Beck C. Nursing research: Principles and methods. 7th ed. Philadelphia: Lippincott, Williams & Wilkins; 2004.

[29] International Labour Organization. International Standard Classification of Occupations: Structure, Group Definitions and Correspondence Tables. Geneva: International Labour Organization; 2008.

[30] Maanoosi M. Stroke rehabilitation in low resource countries: time to provide an organised service. SSMJ. 2024 Feb 19;17(1):27–31. doi: 10.4314/ssmj.v17i1.6

[31] Sahely A, Giles D, Sintler C, Soundy A, Rosewilliam S. Self-management interventions to improve mobility after stroke: an integrative review. Disabil Rehabil. 2023 Jan 2;45(1):9–26. doi: 10.1080/09638288.2022.2028019 35068313

[32] Hughes CML, Padilla A, Hintze A, Raymundo TM, Sera M, Weidner S, et al. Developing an mHealth app for post-stroke upper limb rehabilitation: Feedback from US and Ethiopian rehabilitation clinicians. Health Informatics J. 2020 Jun;26(2):1104–17. doi: 10.1177/1460458219868356 31566456

[33] Hasson F, Keeney S, McKenna H. Research guidelines for the Delphi survey technique. J Adv Nurs. 2000 Oct;32(4):1008–15. doi: 10.1046/j.1365-2648.2000.01567.x 11095242

[34] Whitman NI. The committee meeting alternative. Using the Delphi technique. J Nurs Adm. 1990 Jun-Aug;20(7–8):30–6. doi: 10.1097/00005110-199007000-00008 2199631

[35] Green B, Jones M, Hughes D, Williams A. Applying the Delphi technique in a study of GPs’ information requirements. Health Soc Care Community. 1999 May;7(3):198–205. doi: 10.1046/j.1365-2524.1999.00176.x 11560634

