# Supplementary material for: Self-rehabilitation strategy for rural community-dwelling stroke survivors in a lower-middle income country: a modified Delphi study
Source: PLoS One. 2025 Feb 25;20(2):e0303658. doi: 10.1371/journal.pone.0303658 (PMC11856556; doi:10.1371/journal.pone.0303658)
Supplement: S1 File — (DOCX) [file pone.0303658.s001.docx]

**Challenges in performing daily activities post-stroke: FGD interview guide**

**Ground rules**

- The most important rule is that only one person speaks at a time. There may be a temptation to jump in when someone is talking but please wait until they have finished.
- There are no right or wrong answers
- You do not have to speak in any particular order
- When you do have something to say, please do so. There are many of you in the group and it is important that I obtain the views of each of you
- You do not have to agree with the views of other people in the group
- Does anyone have any questions?
- OK, let’s begin

**Warm up**

- First, I’d like everyone to introduce themselves. Can you tell us your name?

**Introductory question**

I am just going to give you a couple of minutes to think about how your life changed after suffering stroke in terms of how you carryout daily activities within your community.

**Guiding questions**

- How would you describe your current situation?
- Could you mention the various daily activities you do?
- Could you describe the difficulties you encounter in performing your daily activities?
- Explain how you cope with daily needs of personal care such as bathing, dressing, toileting etc
- How do you use your hand(s) to eat, drink water, etc?
- How best do you sit, stand and walk?
- Are there typical cultural-based approaches to support you in carrying out any of the activities mentioned?

**Concluding question**

- Of all the things we’ve discussed today, what would you say are the most important issues you would like to express regarding your experience with daily activities as a stroke survivor?
- Is there any thing anyone wants to add?

**Conclusion**

- Thank you for participating. This has been a very successful discussion
- Your opinions will be a valuable asset to the study
- We hope you have found the discussion interesting
- If there is anything you are unhappy with or wish to complain about, please contact the principal investigator or the supervisor through the contacts provided in the consent forms.
- I would like to remind you that any comments featuring in this report will be anonymous
