# Supplementary material for: Self-rehabilitation strategy for rural community-dwelling stroke survivors in a lower-middle income country: a modified Delphi study
Source: PLoS One. 2025 Feb 25;20(2):e0303658. doi: 10.1371/journal.pone.0303658 (PMC11856556; doi:10.1371/journal.pone.0303658)
Supplement: S3 File — (DOCX) [file pone.0303658.s003.docx]

**DEVELOPMENT OF A SELF-REHABILITATION INTERVENTION MODEL FOR FUNCTIONAL MOBILITY AMONG COMMUNITY-DWELLING STROKE SURVIVORS IN LMICs**

***Delphi Invitation Letter/Information Sheet***

Dear Sir,

My supervisor and I would like to invite you as an expert in the field of rehabilitation for people living with stroke to participate in a Delphi study which is part of a PhD thesis. The aim of the study is to recognize experts’ opinions on a task-specific training model that can be self-administered among community-dwelling stroke survivors in Nigeria.

The model consists of set of trainings that were itemized based on the tasks that were identified as common and challenging to perform by stroke survivors during focus group discussions with community-dwelling stroke survivors in Northern Nigeria. The trainings are designed in a way that can easily be self-administered within the Nigerian community setting. The training activities will be developed in audio-visuals, with an individual demonstrating and verbally describing the activities for stroke survivors to follow and would be administered using tele-rehabilitation technique.

As an established expert in this field we are keen to gain your views on how relevant, appropriate and adequate is each training item in the model. The Delphi Process will consist of 3 rounds. As an expert you are expected to send your answers by e-mail, filling in the specially prepared forms. This Delphi process will involve rating and expressing your opinion on the importance and relevance of each item (round 1). The round 2 of this expert consultation will involve you and other experts following aggregating research relevant diverse and shared opinions in a questionnaire. As in round 1 the focus is to receive feedbacks that will aid final development of the set phenomenon (task-specific training model relevant for self-administration). The round 3 is to invite experts to consider their scores based on group responses in relation to overall responses received and decide/suggest what they deem appropriate between their rating and average opinion of other respondents.

We realize that the professionals we are approaching are extremely busy in their respective fields, but because of the important input you can bring to the project we hope that you will agree to participate in it. In practical terms, we would require no more than two hours of your time, spread out over to three separate occasions. This is a modified Delphi study of two packages one for the upper extremity and two for the lower extremity.

All participating experts will be acknowledged in the final report and will receive a final copy of the model. We would appreciate your participation in this important study that will promote stroke rehabilitation in Sub-Saharan Africa.

If you agree, please complete the attached form and return it by email to **tok2rabs@gmail.com**. Should you wish to discuss the project or your participation in more detail please feel free to contact me on **+2348188763871**, **tok2rabs@gmail.com** or my supervisors on **+2347033318835**, [**iulawal.pth@buk.edu.ng**](mailto:iulawal.pth@buk.edu.ng)**; +46762397653,** conran.joseph@gmail.com

Thank you in advance for your time,

Yours sincerely,

**Rabi’u Ibrahim (Principal investigator)**

Department of Medical Services, Physiotherapy Division, National Assembly Clinic, Abuja Nigeria.

Supervisor:

**Isa U. Lawal (PhD)**

Department of Physiotherapy, Faculty of Allied Health Sciences, College of Health Sciences, Bayero University, Kano, Nigeria.

**DEVELOPMENT OF A SELF-REHABILITATION INTERVENTION MODEL FOR FUNCTIONAL MOBILITY AMONG COMMUNITY-DWELLING STROKE SURVIVORS IN LMICs**

**Principal Investigator:** Rabi’u Ibrahim

**Supervisor:** Dr Isa U. Lawal

**Co-supervisor:** Dr Conran Joseph

**Participant Identification Number for this project:**

**By signing this form, you agree to the followings:**

1. I confirm that I have read and understand the information sheet explaining the above research project and I have had the opportunity to ask questions about the project.

1. I understand that my participation is voluntary and that I am free to withdraw at any time without giving any reason and without there being any negative consequences. In addition, should I not wish to answer any particular question or questions, I am free to decline.
2. I give permission for my anonymous responses to be used during the Delphi process, and to be accessed by members of the research team. I understand that my name will not be linked with the research materials, and I will not be identifiable during the Delphi survey or in the reports that result from the research.
3. I agree to take part in the above research project.

___________________

Name of Participant Date Signature


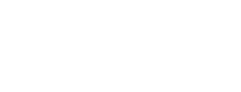


Rabi’u Ibrahim

hhfh

Principal Investigator

Date Signature

**Completion:** *Please return electronically completed forms via email to:* ***tok2rabs@gmail.com***

**Further information:** *Please do not hesitate to contact the principal investigator if you have any concerns or questions. Contact details: Tel:* **+***2348188763871, email:* [***tok2rabs@gmail.com***](mailto:tok2rabs@gmail.com)***,*** or my supervisors on **+2347033318835**, [**iulawal.pth@buk.edu.ng**](mailto:iulawal.pth@buk.edu.ng)**; +46762397653,** conran.joseph@gmail.com

**Copies:** *Please retain a copy of the completed consent form for your personal records.*
