# Supplementary material for: Self-rehabilitation strategy for rural community-dwelling stroke survivors in a lower-middle income country: a modified Delphi study
Source: PLoS One. 2025 Feb 25;20(2):e0303658. doi: 10.1371/journal.pone.0303658 (PMC11856556; doi:10.1371/journal.pone.0303658)
Supplement: S4 File — (DOCX) [file pone.0303658.s004.docx]

**DELPHI INTRODUCTION/INSTRUCTIONS**

The aim of this Delphi study is to develop and validate a suitable Task-specific, Self-rehabilitation Training (TASSRET) intervention model that can be self-administered by the Hausa community-dwelling stroke survivors.

The model consist of set of trainings that were itemized based on the tasks that were identified as common and challenging to perform by stroke survivors during focus group discussions with community-dwelling stroke survivors in Northern Nigeria. The trainings are designed in a way that can easily be self-administered within the Nigerian community setting.

This Delphi process will involve rating and expressing your opinion on the importance and relevance of each item (task) in the TASSRET model. There are two columns for rating and comments for each item. In the comment score you may wish to write any observation regarding the task.

The item ratings will be on a 4- point scale to avoid a neutral and ambivalent midpoint. The rating interpretation is as follows: 1 = not relevant, 2 = somewhat relevant, 3 = quite relevant and 4 = highly relevant. After all the content experts have finished the ratings, the responses will be retrieved and the computation of the item validity will be as follows: for each item, the number of experts giving a rating of 3 or 4 divided by the total number of experts will be computed. A content validity score for item of ≥ 0.78 will be accepted for item to be relevant. Any item that score < 0.78 will either be modified or removed.

Note: The number of repetitions of task in a training session will be included after an ongoing systematic review to determine the adequate number of repetitions of training that can promote motor learning with minimal adverse effect post-stroke.
