# Supplementary material for: Self-rehabilitation strategy for rural community-dwelling stroke survivors in a lower-middle income country: a modified Delphi study
Source: PLoS One. 2025 Feb 25;20(2):e0303658. doi: 10.1371/journal.pone.0303658 (PMC11856556; doi:10.1371/journal.pone.0303658)
Supplement: S5 File — (DOCX) [file pone.0303658.s005.docx]

**ACTIVITIES FOR UPPER EXTREMITY**

**Warm up**

**1**

In a seated position lace your fingers together and then, make large circular movements. You can use your non-affected arm to guide your affected arm. Make 10 slow controlled circles.

Suggestions:

AA: The instruction needs to be clear for instance ‘in an upright seated position’ or ‘lace your fingers with your upper limb straight forward’ or ‘make large circular movements in the horizontal plane’

NV: Specify movement, Circular movements of what? The wrist or what? Are the elbows and shoulders also moving or stabilized?

**SA:** This is not clear enough as to whether this is for the whole arm or for the hands

SN: Is it task specific?

**WS:** This is a very good exercise to start with because the interlacing of fingers creates/stimulates an awareness of the affected arm. Perhaps it needs stated more directly whether the circles involve only wrist or full shoulder movement?

**Correction:** In an upright seated position, lace your fingers with your upper limb straight forward and make large circular movements in the horizontal plane involving full shoulder movements. You can use your non-affected arm to guide the affected arm. Make 10 slow controlled circles.

**2**

Start with your elbow on a table with your arm bent at 90 degrees. Then, curl your arm up just a little, and then release it back down just a little. Slowly repeat 10 times.

Suggestions:

AA: I think the degree of angulation should relate to the joint at which the movement may occur. Also, activity is not quite clear; it may need to be broadened.

NV: Specify – curling (moving hand towards shoulder). I think for all the exercises it would help to explain purpose of the exercise.

**SA:** Would you do this for both arms or just the affected one-why just a little-do you not want as full a range as possible?

SN: To do this exercise, the arm should be attached to the trunk in order not to stimulate flexor synergy of the affected arm.

Is it task specific? Does it have related to daily activity?

The exercise is a kind of movement not activity or task.

**UC:** No indication of starting position (standing or sitting or leaning). I believe sitting though

**WS:** Suggest adding “curl your fist and forearm towards your body”

**Correction:** In an upright seated position, place your elbow on a table with your forearm bent at 90 degrees at the elbow joint and supinated, and the arm attached to the trunk. Then curl your hand and forearm towards your shoulder, and then release it back down. Slowly repeat 10 times.

**5**

Place your affected hand on the table with your palm down. Then, use your non-affected hand to slide your hand to the left and then to the right. Focus on initiating the movement solely from your wrist. Repeat slowly for a total of 10 repetitions.

Suggestions:

NV: If they have grade 2 muscle strength they may benefit from doing some of the exercises active even during warm up.

*See previous comment about doing some exercises active. It looks like most if not all of these warm up activities are passive because emphasis is not on them doing the exercise and assisting with unaffected – seem to emphasize using unaffected to move (passive and not even leaning towards active assisted) – can do gravity eliminated positions for get more out of the movements considering grade 2.*

SA: Are you saying that the unaffected hand should assist the affected one-if so then make this clearer

SN: Also swipe forward and backward while arms are attached to the trunk

**Correction:** In an upright seated position, place your affected hand on the table with your palm down, then slide your hand to the left and then to the right at the wrist joint. Focus on initiating the movement solely from your wrist. You may use the unaffected hand to assist in moving the affected hand.

**A: Trainings for reaching**

**Instruction:** *Where the use of an object is involved, the initial position of the object should be about 15cm (0.15m) from you in all training, and start with 200 repetitions of each task in a session. After every week increase the distance of the object by 7.5cm (0.075m) and the number of repetitions by 10% (20 repetitions).*

Suggestion:

NV: I support the increase in distance and repetition per week as a matter of principle however, 200 repetitions is too much. What is this number based on? With 20 reps increase per week it means a person will do 300 by week 5 or 6 – is this the number of repetitions that people do in clinical practice, per activity? **(**Is it safe to assume that the that the starting position is the same as the one used for warm up)

SA: Very high repetition rate?

SN: Please set a maximum value for reaching distance.

What about stroke survivors who is not able to extend fully his arm? I recommend to use light splint to maintain affected elbow in extension in these situations.

Number of repetitions are so high!

**SW:** It should be clearly stated whether each task could be divided into sets, or it’s a continuing task of 200 times.

Correction: *Positioning for the activities in this section has the patient seated upright in a chair with a firm back and no armrests, and the trunk contacting the back of the chair. The head should be in a neutral position, with feet in contact with the floor and the hands resting on the lap.*

*The subject sits close to a table, within 15cm (0.15m) distance from the anterior torso to the front edge of the table. Where the use of an object is involved, the initial position of the object should be about 30cm (0.3m) from the anterior torso in all training, and start with 10 repetitions of each task in a session. After every week increase the distance of the object by 7.5cm (0.075m) till you reach a maximum distance of 50cm (0.5m), and the number of repetitions be increased by 50% of the starting number of repetitions weekly (5 repetitions).*

Objects to be used may include bottle of water and cups of different sizes etc.

**1**

Stretch out the affected arm to reach and touch an object on a table directly in front of you

Suggestions:

AR: Items 1-3 can be combined into one

NV: Include instructions about position of the trunk or avoidance of any possible trick movements (this applies to all) – need to make sure they move the upper limb and not compensate a lot with trunk movement – this has to be emphasized and again, use of pictures will be very useful

Progression – general instructions at the beginning are more about number of repetitions and distance of object from the body. Now speed is brought up but there is no reference point. What should they consider acceptable speed? Same comment applies to all categories.

**2**

Stretch out the affected arm to reach and touch an object on a table placed on the affected side

SA: Make it clearer as to where the table is-that is how far away it must be

WS: Very important to stimulate neural pathways on the affected side.

**3**

Stretch out the affected arm to reach and touch an object on a table placed on the unaffected side

**SA:** Make it clearer as to where the table is-that is how far away it must be

WS: Very important

Correction: In an upright seated position, stretch out the affected arm to reach and touch an object on a table directly in front of you, make sure you move the upper limb and not compensate a lot with trunk movement. Do same task with the object placed at about 15cm from its original position on affected side and then on the unaffected side. Using both hands do the same tasks

**5**

Lift up the affected arm to reach and touch an object hanged above your head

SA: Do you want them to maintain a good posture while doing this

SN: During this exercise, the affected arm should be attached to the trunk in order not to use trunk compensatory movements.

SW: It should be hung in the front in a visible position so that the patients can clearly see the object.

WS: Perhaps clarify… e.g. don’t use your unaffected arm to lift.

Correction: Using the affected hand, reach and touch an object hanged at a height of about 15cm in front of you in a visible position. Using both hands do the same task.

**6**

With the affected hand touch the unaffected shoulder and return your hand to the initial position

AR: Does not seem like a separate exercise

NV: Returning to initial position – what is the initial position for this exercise. Please explain starting point clearly for all exercises to leave no room for assumptions.

I don’t understand the second part of the progression: “move the hand from the shoulder across the arm and forearm to the back of the hand” – this is where pictures will be helpful

SN: Please relates it to the function or a goal.

WS: Midline crossing is really important.

**7**

Using the affected hand touch your head and return your hand to the initial position

SA: Again what about posture-so that person remains sitting upright.

Correction: Using the affected hand touch your head and return your hand to the initial position (see starting position above under instruction)

**8**

With both hands touch your shoulders at once

NV: Explain starting position that they go back to as they do they do repetitions

SA: Maybe all of the above exercises could be done with both hands-specially to start with

TH: How? Please explain

Correction: With both hands touch your shoulders at once (each hand to the opposite side shoulder) crossing the forearms. Then return your hand to the initial position (see starting position above under instruction)

**B**: **Training for grasp/Grip**

**1**

Place hand around object, try and squeeze object, then lift your fingers/thumb away from the object

NV: Speed is not key here but you can add if you feel strongly about it - what is more important is the size of the object. Rather focus progression on moving from bigger to smaller object. The adaptation just refers to using different size… - make it very clear that they should start with bigger progressing to smaller

SN: Do you mean pick up instead of place and squeeze? Can you squeeze any object for instance

TH: What type of object am I squeezing my hand around?

WS: Squeeze for how long?

**2**

Place hand around object, try and squeeze object, lift it up from the table, then place it back and lift your fingers/thumb away from the object

NV: The last part of exercise description is not clear: “…then drop it back and lift your fingers/thumb away from the object”

Why is lifting fingers from object done after dropping it back – this is not clear. If they can drop it there is no need to lift fingers. If they can’t drop it means they are still at the same level as first exercise. Make this very clear. I am not sure if you are trying to introduce grasp with arm elevation or trying to introduce use of gravity to help with release of object from the hand. Please think about this and make the instructions and purpose very clear

SA: How far must person lift the object?

Correction:

Place the affected hand around an object, lift it up to about 15cm from the table, then place it back and lift your fingers/thumb away from the object and return to the starting position.

**4**

Stretch out the affected arm to reach an object (e g water bottle) placed on the floor by the affected side, at about a 15cm away from the chair you are seated on, then hold the object between fingers and thumb, release it and return to the starting position.

SA: See precautions above re beding forward to floor

SW: Because this is intended for patients to practice at home, it is preferable to make it clear where the object is on the floor? This step requires a good way to keep their balance on the seats. Which degree of sitting balance should patients be needed to maintain?

**C: Training for moving objects (AR:** This can be included in the section above, grip and moving objects can be combined)

**1**

Place hand around an object, hold it between fingers and thumb, lift it up from the table and transfer it to another position then pick it again and return it to its initial position

NV: Comment about increasing distance of reach is the key outcome and should be emphasized more than speed. Same applies to all upper limb tasks. Speed is important but it must be very clear that quality of movement and number of repetitions (endurance) and distance is relatively more important than speed

Activity: ‘transfer it to another position then pick it again’ – make it clear that it is another position on the same level unless this is not the case. Then specify.

SA: Make it clearer as to where the other position is\

WS: Another position where?

Correction: Place the affected hand around an object, hold it between fingers and thumb, lift it up from the table and move it to about 30cm towards the affected side. Then pick it again and return it to its initial position. Do the same towards the unaffected side.

**2**

Using the affected hand lift an object (e g water bottle) from the table and place it on the floor by the affected side and return to starting position. Then pick the object and place it on the table in its initial position.

**SA:** Define higher and lower so that person understands what you mean

Correction: Using the affected hand lift an object (e g water bottle) from the table and place it on the floor by the affected side and return to starting position. Then pick the object and place it on the table in its initial position.

**3**

Take lid of a bottle or a jar and return it in place

NV: Emphasis should be on progression to bigger lid to smaller lid if this is about rolling the lid off the jar and not the type that is just for lift it off (I hope my point is clear – depends on the type of lid:

Correction: progress from bigger to smaller lid

**4**

Open a food bowl, place the cover down and then replace it back

NV: I cannot give a rating for this one because I don’t know what is meant by ‘food bowl’ here.

SA: Not sure what is meant by a food bowl with a cover

Correction:

**5**

Using both hands pick up a plate and transfer it to another position

NV: Also include empty plate and progress to plate with food

Specify the other position here – is the progression from the same level (horizontal) to later doing vertical. Has to be very clear what is considered progression for the patient.

Correction: Using both hands pick up a plate and transfer it to another position (see instruction in the beginning of this section about positioning and using an object)

**6**

With the affected hand take a cup to your mouth.

NV: Is the use of cup with handle a progression from using a cup without handle or are they using cup with handle from beginning to end? Make it very clear. My understanding is that you are moving from full grasp to e.g. pincer grip – leave no room for assumptions

SA: What if this is not the person’s dominant hand-do you not want normal movement and activities

Correction: With the affected hand take a cup to your mouth (see instruction in the beginning of this section about positioning and using an object)

**D: Training for object manipulation**

**1**

Use both hands to fold and unfold a piece of cloth

NV: Need an activity which will make it impossible without use of affected hand – they can compensate a lot here if using both hands unless progression includes use of affected hand only

WS: Important for coordination.

Correction: Use both hands to fold and unfold a piece of cloth placed on a table.

**2**

Open covered pots of different sizes and transfer any powdered substance to a cup with a spoon, then close the pot

AA: May I suggest, this item to be rephrased as thus ‘open covered pots (containing any form of powdered substance) of different sizes and transfer the powdered substance into a cup with a spoon, then close the pot

NV: Open pot with affected or unaffected. Same applies to transfer – is this transfer about scooping out the powdered substance. Please make this clear. I am not able to rate because I don’t understand what patient is asked to do in relation to affected vs unaffected upper limb

SA: Do you mean jars-eg of sugar etc.

WS: Important for coordination.

Correction: Use the non-dominant hand to open covered containers (containing any form of powdered substance, eg jars of sugar etc) of different sizes and use the dominant hand to transfer the powdered substance with a spoon into a cup, then close the pot.

**3**

Open a box, pick up objects inside the box, and transfer them to a pot, then close the box

**AA:** Here, too, we may add ‘open a box (containing some objects), pick up the objects and transfer them to a pot, then close the box

NV: Specify: which hand does what. The activity can be achieved without much use of affected hand. I support all these activities as a matter of principle provided the instructions are such that most of the movements will be done with the affected hand and that it will not just be used to stabilize but to manipulate – this comment applies to all items in this category

WS: Important for coordination and planning.

Correction: Use the non-dominant hand to open a box (containing some objects), and use the dominant hand to pick up the objects and transfer them to a pot, then close the box.

**4**

Pick up coins and small stones on the table or from the floor, and put the stones in a pot and gather the coins

NV: Indicate that picking from floor is progression from picking on table. They must not stop at table activity or if they can do floor activity they will not have to do table activity as a matter of principle for progression

SA: Again precautions re leaning forward to floor

WS: Important for fine motor coordination.

Correction: Using the affected hand pick up coins and small stones on the table, put the stones in a pot placed on the table and gather the coins.

Progression: do same with the pot placed on the floor.

**5**

Open a box with key, pick up objects inside the box, and transfer them to a pot, then lock the box

**AA:** Here, too, we may add ‘open a box (containing some objects), pick up the objects and transfer them to a pot, then close the box

NV: This is progression of 3 with addition of unlocking box – consider combining or removing picking of object here (repetition)

Thickness of key – specify that they start with relatively thick key and progress to thin and small one.

Correction: Lock and unlock a box with a key.

Progression; start with thicker keys then progress with thinner keys.

**6**

Pick up and transfer jars, bottles, and cups of different sizes and weights located on a table or from the floor. Transfer the liquid contents from jars and bottles to cups

**NV:** A combination of two activities – the focus should be on transfer of liquids – the pickup and transfer of objects was done in movement of objects.

SA: Again I assume that you require normal movements from people so they would do these activites in the way that they have always done them.

Correction: Transfer the liquid contents from jars and bottles to cups. Involve both hands and do normal movement as much as possible.

**7**

Take money in and out of the pocket using the affected hand

SA: Again I assume that you require normal movements from people so they would do these activites in the way that they have always done them

TH: Money in coins or note?

**WS:** Similar to 4. But functionally important.

Correction: Take money (notes) in and out of the pocket using the affected hand

Progress: do the training using different pockets on your cloth.

**8**

Use both hands to pick up cap and pace it on your head

AA: I am sure is place you wanted to write here not pace

**NV:** Why both hands – should be affected hand

SA: What if person does not wear a cap-is there an alternative

TH: Is this applicable to males only?

**WS:** “place” – functionally important.

Correction: Use the affected hand to pick up cap and place it on your head, then use both hands to adjust it well. For women use both hands to tie a head tie on your head.

**9**

Use both hands to tie wrapper around your body

AA: Can this item be applicable to the men folk considering this environment?

NV: Is this gender specific or applicable to both men and women

**TH:** Is this applicable to females only?

Correction: Use both hands to tie wrapper around your body (for women)

**E: Training for hand/fingers precision**

**3**

Use both hands to button and unbutton your shirt

WS: Very functionally important.

**4**

Use the affected hand to press numbers on a phone with the index finger

SA: Again see comments re normal movements

TH: Please what type of phone?

Correction: Use the non-dominant hand to hold a mobile phone and press numbers and/or typh texts with the dominant hand.

**ACTIVITIES FOR THE TRUNK**

**Warm up**

**2**

From a seated position, slowly bend your trunk to the right side and then to the left side, then, return to an upright.

SA: Suggest this is done slowly

TH: I suggest, “from the upright sitting position, bend to the right, come to upright, then bend to the left and come back to the upright”.

Correction: From the upright sitting position, bend to the right, come to upright, then bend to the left and come back to the upright (do it slowly).

**3**

From a seated position, turn your trunk to the right side and then left side facing your back side. Be sure to keep your spine straight and don’t twist to the point of pain.

NV: The part about “facing your back side” is not clear. I don’t understand what is expected here. The trunk rotation to left and right is clear

**SA:** Suggest this is done slowly

TH: Please always ask the patient to start from the midline then right/left then back to midline then left/right. Instructions must be clear and simple

**WS: “turn/twist…”**

Correction: From the upright sitting position, rotate your trunk to the right, then return to the midline and rotate to the left (do it slowly).

**A: Training for Trunk Strength (**I would suggest trunk strengthening exercises are incorporated into functional activities?)

**2**

Clasp your hands together, and then punch forward while keeping your arms parallel to the floor. Use your back muscles to come back up.

AR: Is this for the trunk or for UL mobility? Should be a 4 for UL mobility and 2 if for the trunk

**NV:** I don’t understand this activity. Initially I thought it is about punching forward and the parallel to the floor even confirmed my understanding. I then got confused by the ‘use of back muscles to come up – coming up from what position??

My rating is based on understanding that they remain in an upright seated position while punching

**SA:** Not clear as to what you mean here

**TH**: “Punch forward” is not clear to me.

**WS:** Perhaps clarify “come back up”

Correction: From the upright sitting position bend your trunk forward while extending the dominant hand to reach an object place on a table at a distance of about 60cm (initially), and come back to the upright sitting position (avoid using the arms to assist in either bending or coming back to the upright position as much as possible).

**ACTIVITIES FOR BALANCE**

**Warm up**

**1**

Hold onto the chair or counter, and raise yourself up onto your tiptoes, keeping your knees straight and holding your upper body tall. Lower yourself back to the floor slowly, and repeat.

NV: Rating is based on assumption is that this is for a person preparing for standing balance activities

**SN:** Is it performed in standing position?

**TH:** Please indicate the Starting position

Correction: In a standing position, hold onto the chair or counter, and raise yourself up onto your tiptoes, keeping your knees straight and holding your upper body tall. Lower yourself back to the floor slowly, and repeat.

**4**

Place your back against a wall, standing tall. Slowly lower into a squatting position, holding on with one hand if needed or not holding on at all. Move up to a standing position and repeat.

NV: Leaning against any form of support structure takes away from the balance reeducation component. This is just a strengthening exercise for the lower limbs which will translate into good balance –

**ACTIVITIES FOR LOWER EXTREMITY**

**Warm up**

**5**

Start with your affected leg still crossed over your other leg. Then, flex your foot back towards your shin – a movement known as dorsiflexion. If you cannot do this, use your hand to assist your foot through the movement. Repeat 10 times.

NV: Why do you say ‘still crossed’ – when was it crossed

Not clear what this is supposed to achieve – if they can’t dorsiflex, asking them to lean forward to assist foot is even more challenging to balance – I don’t understand why it is presented as something they should do if they struggle. If they can’t dorsiflex because of weak e.g. tibialis anterior) then that is purely a lower limb strength problem and asking them to lean forward to do passive or active assisted dorsiflexion in sitting will challenge their balance far more the original step – that is why I don’t understand what the goal of this next step is in relation to balance, not ankle movement

**TH:** What is the starting position?

**Training for Balance**

**4**

Place your back against a wall, standing tall. Slowly lower into a squatting position, holding on with one hand if needed or not holding on at all. Move up to a standing position and repeat.

**NV:** Leaning against any form of support structure takes away from the balance reeducation component. This is just a strengthening exercise for the lower limbs which will translate into good balance –

**ACTIVITIES FOR LOWER EXTREMITY**

**Warm up**

**5**

Start with your affected leg still crossed over your other leg. Then, flex your foot back towards your shin – a movement known as dorsiflexion. If you cannot do this, use your hand to assist your foot through the movement. Repeat 10 times.

NV: Why do you say ‘still crossed’ – when was it crossed

Not clear what this is supposed to achieve – if they can’t dorsiflex, asking them to lean forward to assist foot is even more challenging to balance – I don’t understand why it is presented as something they should do if they struggle. If they can’t dorsiflex because of weak e.g. tibialis anterior) then that is purely a lower limb strength problem and asking them to lean forward to do passive or active assisted dorsiflexion in sitting will challenge their balance far more the original step – that is why I don’t understand what the goal of this next step is in relation to balance, not ankle movement

**TH:** What is the starting position?

**C: Trainings for Transfers from sit to stand**

**2**

Stand up from a sitting position on the edge of bed with the support of the unaffected hand

No suggestion

**3**

Sit-to-stand from chair by placing the affected foot behind.

**NV:** Indicate that they reduction of seating level height and not necessarily chair but any surface also including compliant surface such as bed and sofas/couch and doing it with and later without use of upper limb for support.

**TH:** “..placing the affected foot behind”? Not clear to me.

Correction: From an upright sitting position (on a chair) stand up by placing the unaffected foot forward until you stand upright. Stay for about 30 seconds and sit back.

Progression: use shorter stool.

**D: Training for Maintaining Standing Position**

**1**

In standing position lift the affected leg sideways with support nearby

SA: Make it clearer as to how the leg must be lifted sideways

TH: What should be the height of the block?

Correction:

**2**

Rise and lower yourself from a high surface (e.g. a block) with support nearby

NV: The rise and lower is not clear – is this heel raises of simulation of sit to stand – please clarify. I am not able to rate as I don’t understand the activity

**SA:** Not sure what you mean-where would person find a block that is a high surface

TH: How high should the surface be at the beginning?

**WS:** Do you mean step up and step down?

Correction: Step up and down a surface of about 5cm height (at the start).

Progress to using higher surfaces to a maximum of 25cm high.

**3**

While standing on the unaffected leg place the affected leg on a bottle (or any hard cylindrical object) then roll forwards and backwards with support

**NV**: This is beneficial but what is needed is more of ability to carry weight through affected leg while moving unaffected leg – use of antigravity muscles rather than open kinematic chain activity for affected leg unless this is preparation for swing phase of gait

**TH:** “Roll forwards and backwards”?

Correction: While standing on the affected leg place the unaffected leg on a bottle (or any hard cylindrical object) then roll the unaffected leg forwards (to about 5cm distance) and backwards. You may hold unto a support and/or a family caregiver should be around to provide support when and if necessary.

**E: Training for Reaching in Standing**

**1**

While in standing position reach an object in front of you with the affected hand

**AA**: Let’s use ‘the affected hand’ for uniformity in progression

**NV**; Reduction of hand support? When was hand support encouraged – are they not supposed to do it without hand support from the onset?

What is the primary purpose of activities in this category – are they for upper limb function of for a combination of standing balance and upper limb function. If a combination some activities will have to focus primarily on balance and other on upper limb function

**TH**: Should the object being reached for be at the height level or above the shoulder level of the patient?

Correction: While in standing position use the affected hand to reach an object placed at the level of your shoulder (initially) at about 30cm in front of you.

Progression: place the object above the shoulder level.

**3**

Stand and reach for objects placed in varying positions and heights, such as low stool, high shelf, to the side

NV: Performing with paretic limb on step – not clear

If activities within this category are to improve dynamic standing balance, emphasis should be on reaching towards affected side and increasing distance of reach. Can also reach with unaffected side but still towards affected side to increase weight bearing through affected leg.

I don’t see this being emphasized in this section (E) and this makes me wonder whether the focus is on UL or balance. UL has been covered a lot in the other document. There is a need to focus more on balance while taking into consideration

**SA:** Are these not progressions of the above-so could be written as one exercise

**TH**: Remember that to reach for an object on the stool while a patient is in standing position means the patient would have to bend forward. How about safety from fall etc?

**WS:** Very functionally important

Correction: In standing position reach for objects positioned at about 30cm away from you on the affected side, and at the level of your shoulder (initially).

Progression: place the object above the shoulder level, also use the unaffected hand to reach the same object at the same position.

**F: Training for Stepping and Walking**

**1**

In standing, lift up the affected leg and place foot forward to marks on the ground aiming for control and accuracy

**NV**: More emphasis for all activities in this section should be on minimizing use of upper limb for support

**SW:** Because this is intended for patients to practice at home, it is preferable to make it clear where the object is on the floor? How far should the marker be placed in front of the patient?

Correction: In standing position, take a step forward with the affected leg and place foot on step marks on the ground at an interval of about 70 to 80cm (for males) and 60 to 70cm (for females) aiming for control and accuracy.

**2**

Take Step forward to a mark on the floor in front

**SA**: Make it clear which leg you mean

SW: Because this is intended for patients to practice at home, it is preferable to make it clear where the object is on the floor? How far should the marker be placed in front of the patient?

**TH:** What is the distance in front to which the patient steps ?

**3**

Step forward onto a step with the paretic limb

**SA:** Why are you now calling the affected leg the paretic leg-your terminology should stay the same

**SW**: Same as 1

Step height should be specified.

**TH:** Step onto what step? On a staircase or a low stool/platform in front of him? To ensure that any one, anyone from any part of the globe can easily use this instrument, make your instructions and settings very clear to avoid confusion.

**4**

Step up onto a step, starting with the affected leg, and step down, starting with the non-affected leg

**SA:** Make it clear that they should start this exercise by holding onto something safe

SW: Step height should be specified.

Correction: Step up onto a step (by holding onto something safe), starting with the affected leg, and step down, starting with the non-affected leg.

Progression: Step up with non-affected first, then step down with the affected leg, and use less support.

**5**

Walk over-ground stepping on marked points

**SW:** Same as 1

**TH:** Start this adaptation indoor before outdoor.
