# Supplementary material for: Self-rehabilitation strategy for rural community-dwelling stroke survivors in a lower-middle income country: a modified Delphi study
Source: PLoS One. 2025 Feb 25;20(2):e0303658. doi: 10.1371/journal.pone.0303658 (PMC11856556; doi:10.1371/journal.pone.0303658)
Supplement: S6 File — (ZIP) [file pone.0303658.s006.zip › S6 Delphi 1 responses/P10.docx]

**TASK-SPECIFIC SELF-REHABILITATION TRAINING (TASSRET) FOR COMMUNITY-DWELLING STROKE SURVIVORS**

**ACTIVITIES FOR UPPER EXTREMITY**

**Warm up**

|  | **Training** | **Rating** | **Comments** |
| --- | --- | --- | --- |
| **1.** | In a seated position lace your fingers together and then, make large circular movements. You can use your non-affected arm to guide your affected arm. Make 10 slow controlled circles. | **3** | **Specify movement, Circular movements of what? The wrist or what? Are the elbows and shoulders also moving or stabilized** |
| **2.** | Start with your elbow on a table with your arm bent at 90 degrees. Then, curl your arm up just a little, and then release it back down just a little. Slowly repeat 10 times. | **3** | **Specify – curling (moving hand towards shoulder). I think for all the exercises it would help to explain purpose of the exercise** |
| **3.** | From a seated position, gently prop yourself up on your affected arm about one foot away from your body. Then gently lean into it. You should feel a mild stretch on your affected side.   - If it feels good, hold the stretch for 10 seconds, and then return to center. Repeat on the other side for a total of 3 sets. - If it doesn’t feel good, stop the stretch immediately. | **4** | **Use of pictures for these activities would help because it takes time to figure out what is expected.** |
| **4.** | Place your elbow on a table, and then use your unaffected hand to stretch your affected hand at the wrist. Stretch backward, and then stretch forward. Perform this movement slowly for a total of 5 repetitions. | **3** | **If they have grade 2 muscle strength they may benefit from doing some of the exercises active even during warm up** |
| **5.** | Place your affected hand on the table with your palm down. Then, use your non-affected hand to slide your hand to the left and then to the right. Focus on initiating the movement solely from your wrist. Repeat slowly for a total of 10 repetitions. | **3** | **See previous comment about doing some exercises active. It looks like most if not all of these warm up activities are passive because emphasis is not on them doing the exercise and assisting with unaffected – seem to emphasize using unaffected to move (passive and not even leaning towards active assisted) – can do gravity eliminated positions for get more out of the movements considering grade 2** |

**Trainings**

**A. Trainings for reaching**

|  | **Activity** | **Progression/Adaptation** | **Rating** | **Comment** |
| --- | --- | --- | --- | --- |
|  | ***Where the use of an object is involved, the initial position of the object should be about 15cm (0.15m) from you in all training, and start with 200 repetitions of each task in a session.*** | ***After every week increase the distance of the object by 7.5cm (0.075m) and the number of repetitions by 10% (20 repetitions).*** | **4** | **I support the increase in distance and repetition per week as a matter of principle however, 200 repetitions is too much. What is this number based on? With 20 reps increase per week it means a person will do 300 by week 5 or 6 – is this the number of repetitions that people do in clinical practice, per activity?** |
| **1.** | Stretch out the affected arm to reach and touch an object on a table directly in front of you | *Progression*: Increase the speed and the number of repetitions  *Adaptation*: Increase the distance of the object from you | 4 | Include instructions about position of the trunk or avoidance of any possible trick movements (this applies to all) – need to make sure they move the upper limb and not compensate a lot with trunk movement – this has to be emphasized and again, use of pictures will be very useful  Progression – general instructions at the beginning are more about number of repetitions and distance of object from the body. Now speed is brought up but there is no reference point. What should they consider acceptable speed? Same comment applies to all categories. |
| **2.** | Stretch out the affected arm to reach and touch an object on a table placed on the affected side | *Progression*: Increase the speed and the number of repetitions  *Adaptation*: Increase the distance of the object from you | 4 |  |
| **3.** | Stretch out the affected arm to reach and touch an object on a table placed on the unaffected side | *Progression*: Increase the speed and the number of repetitions  *Adaptation*: Increase the distance of the object from you | 4 |  |
| **4.** | Stretch out the affected arm to reach and touch an object on the floor | *Progression*: Increase the speed and the number of repetitions  *Adaptation*: Increase the distance of the object from you | 4 |  |
| **5**. | Lift up the affected arm to reach and touch an object hanged above your head | *Progression*: Increase the speed and the number of repetitions  *Adaptation*: Increase the distance of the object from you | 4 |  |
| **6.** | With the affected hand touch the unaffected shoulder and return your hand to the initial position | *Progression*: Increase the number of repetitions and move the hand from the shoulder across the arm and forearm to the back of the hand | 3 | Returning to initial position – what is the initial position for this exercise. Please explain starting point clearly for all exercises to leave no room for assumptions.  I don’t understand the second part of the progression: “move the hand from the shoulder across the arm and forearm to the back of the hand” – this is where pictures will be helpful |
| **7.** | Using the affected hand touch your head and return your hand to the initial position | *Progression*: Increase the speed and the number of repetitions and move the hand towards the upper back | 3 |  |
| **8.** | With both hands touch your shoulders at once | *Progression*: Increase the speed and the number of repetitions | 3 | Explain starting position that they go back to as they do they do repetitions |

**B. Training for grasp/Grip**

|  | **Activity** | **Progression/Adaptation** | **Rating** | **Comment** |
| --- | --- | --- | --- | --- |
| **1.** | Place hand around object, try and squeeze object, then lift your fingers/thumb away from the object | *Progression*: Increase the speed and the number of repetitions  *Adaptation*: use different size and shape of the objects | 4 | Speed is not key here but you can add if you feel strongly about it - what is more important is the size of the object. Rather focus progression on moving from bigger to smaller object. The adaptation just refers to using different size… - make it very clear that they should start with bigger progressing to smaller |
| **2.** | Place hand around object, try and squeeze object, lift it up from the table, then drop it back and lift your fingers/thumb away from the object | *Progression*: Increase the speed and the number of repetitions  *Adaptation*: use different size and shape of the objects | 2 | The last part of exercise description is not clear: “…then drop it back and lift your fingers/thumb away from the object”  Why is lifting fingers from object done after dropping it back – this is not clear. If they can drop it there is no need to lift fingers. If they can’t drop it means they are still at the same level as first exercise. Make this very clear. I am not sure if you are trying to introduce grasp with arm elevation or trying to introduce use of gravity to help with release of object from the hand. Please think about this and make the instructions and purpose very clear |
| **3.** | Lift up the affected arm to reach an object hanged above your head, then hold the object between fingers and thumb, release it and put down your arm | *Progression*: Increase the speed and the number of repetitions  *Adaptation*: use different size and shape of the objects | 4 | So you are only focusing on use of fingers for grip and not e.g. cylindrical grip etc. (pictures of various objects that can help with grip/grasp can also help here – can include examples of everyday items that most households would have) examples of objects apply to all: I just remembered to write it here. |
| **4.** | Stretch out the affected arm to reach an object on the floor, then hold the object between fingers and thumb, release it and return to the starting position | *Progression*: Increase the speed and the number of repetitions  *Adaptation*: use different size and shape of the objects | 4 |  |

**C. Training for moving objects**

|  | **Activity** | **Progression/Adaptation** | **Rating** | **Comment** |
| --- | --- | --- | --- | --- |
| **1.** | Place hand around an object, hold it between fingers and thumb, lift it up from the table and transfer it to another position then pick it again and return it to its initial position | *Progression*: Increase the speed, number of repetitions and the distance between the two positions  *Adaptation*: use different size and shape of the objects | 4 | Comment about increasing distance of reach is the key outcome and should be emphasized more than speed. Same applies to all upper limb tasks. Speed is important but it must be very clear that quality of movement and number of repetitions (endurance) and distance is relatively more important than speed  Activity: ‘transfer it to another position then pick it again’ – make it clear that it is another position on the same level unless this is not the case. Then specify. |
| **2.** | Lift an object from a higher level to a lower level and vice versa | *Progression*: Increase the speed, number of repetitions and the distance between the two positions  *Adaptation*: use different size and shape of the objects | 4 |  |
| **3.** | Take lid of a bottle or a jar and return it in place | *Progression*: Increase the speed and the number of repetitions  *Adaptation*: use different size and shape of bottles and jars | 4 | Emphasis should be on progression to bigger lid to smaller lid if this is about rolling the lid off the jar and not the type that is just for lift it off (I hope my point is clear – depends on the type of lid: |
| **4.** | Open a food bowl, place the cover down and then replace it back | *Progression*: Increase the speed and the number of repetitions  *Adaptation*: use different size and shape of food bowl |  | I cannot give a rating for this one because I don’t know what is meant by ‘food bowl’ here. |
| **5.** | Using both hands pick up a plate and transfer it to another position | *Progression*: Increase the speed and the number of repetitions  *Adaptation*: use different size and shape of plates, practice different positions in terms of height level | 4 | Also include empty plate and progress to plate with food  Specify the other position here – is the progression from the same level (horizontal) to later doing vertical. Has to be very clear what is considered progression for the patient. |
| **6.** | With the affected hand take a cup to your mouth | *Progression*: Increase the speed and the number of repetitions and use cups with handle  *Adaptation*: : use different size and shape of cups | 4 | Is the use of cup with handle a progression from using a cup without handle or are they using cup with handle from beginning to end? Make it very clear. My understanding is that you are moving from full grasp to e.g. pincer grip – leave no room for assumptions |

**D. Training for object manipulation**

|  | **Activity** | **Progression/Adaptation** | **Rating** | **Comment** |
| --- | --- | --- | --- | --- |
| **1.** | Use both hands to fold and unfold a piece of cloth | *Progression*: Increase the speed and the number of repetitions  *Adaptation*: use different kind of sheets including piece of cloth, bed sheets etc. | 2 | Need an activity which will make it impossible without use of affected hand – they can compensate a lot here if using both hands unless progression includes use of affected hand only |
| **2.** | Open covered pots of different sizes and transfer any powdered substance to a cup with a spoon, then close the pot | *Progression*: Increase the speed and the number of repetitions  *Adaptation*: Increase the diameter of the spoon | 4  if affected hand open and then scoops | Open pot with affected or unaffected. Same applies to transfer – is this transfer about scooping out the powdered substance. Please make this clear. I am not able to rate because I don’t understand what patient is asked to do in relation to affected vs unaffected upper limb |
| **3.** | Open a box, pick up objects inside the box, and transfer them to a pot, then close the box | *Progression*: Increase the speed and the number of repetitions  *Adaptation*: Increase the size of the objects | 4  If unaffected hand just stabilizes | Specify: which had does what. The activity can be achieved without much use of affected hand. I support all these activities as a matter of principle provided the instructions are such that most of the movements will be done with the affected hand and that it will not just be used to stabilize but to manipulate – this comment applies to all items in this category |
| **4.** | Pick up coins and small stones on the table or from the floor, and put the stones in a pot and gather the coins | *Progression*: Increase both the speed and the number of coins and stones  *Adaptation*: use different sizes of stones | 4 if only affected hand is used | Indicate that picking from floor is progression from picking on table. They must not stop at table activity or if they can do floor activity they will not have to do table activity as a matter of principle for progression |
| **5.** | Open a box with key, pick up objects inside the box, and transfer them to a pot, then lock the box | *Progression*: Increase the speed and the number of repetitions  *Adaptation*: Increase the thickness of the key and size of the objects | 4  If key manipulation is only with affected hand and if picking of objects is removed from activity | This is progression of 3 with addition of unlocking box – consider combining or removing picking of object here (repetition)  Thickness of key – specify that they start with relatively think key and progress to think and small one |
| **6.** | Pick up and transfer jars, bottles, and cups of different sizes and weights located on a table or from the floor. Transfer the liquid contents from jars and bottles to cups | *Progression*: Increase the speed as well as the distance from the object to be reached  *Adaptation*: Reduce the volume of liquid in the jars and bottles | 4  For transfer of liquids | A combination of two activities – the focus should be on transfer of liquids – the pick up and transfer of objects was done in movement of objects. |
| **7.** | Take money in and out of the pocket | *Progression*: Increase the speed and the number of repetitions  *Adaptation*: use different pockets on your clothe | 3 |  |
| **8.** | Use both hands to pick up cap and pace it on your head | *Progression*: Increase the speed and the number of repetitions  *Adaptation*: use different types of caps | 1 | Why both hands – should be affected hand |
| **9.** | Use both hands to tie wrapper around your body | *Progression*: Increase the speed and the number of repetitions  *Adaptation*: use other things like head scarf | 3 | Is this gender specific or applicable to both men and women |
| **10.** | With affected hand pick nuts from a container and break it’s shell | *Progression*: Increase the number of repetitions  *Adaptation*: bring out the nut and place it in another container | 1 | Can one break an nut shell with just affected hand – is this not a bilateral upper limb activity? It is possible that I am thinking of a different type of nuts (with hard shell) – if it is about ‘soft shell’ it is ok |

**E. Training for hand/fingers precision**

|  |
| --- |
|  |

|  | **Activity** | **Progression/Adaptation** | **Rating** | **Comment** |
| --- | --- | --- | --- | --- |
| **1.** | Touch the tip of each finger with the tip of the thumb | *Progression*: Increase the speed and the number of repetitions, also touch the proximal, middle and the distal phalanx of each finger  *Adaptation:* do it without looking at the hand | 1 | Covered through activities in previous sections |
| **2.** | Pick up coins or small stones on the table from a particular point to a marked point | *Progression*: Increase the speed and the number of repetitions  *Adaptation*: use different sizes of stones | 1 | Covered in previous sections |
| **3.** | Use both hands to button and unbutton your shirt | *Progression*: Increase the speed and the number of repetitions  *Adaptation*: do the task without looking | 4 |  |
| **4.** | Use the affected hand to press numbers on a phone with the index finger | *Progression*: Increase the speed and the number of repetitions and use other fingers  *Adaptation*: type text | 4 |  |

**ACTIVITIES FOR THE TRUNK**

**Warm up**

|  | **Training** | **Rating** | **Comments** |
| --- | --- | --- | --- |
| **1.** | From a seated position, press your back against the back rest. | **2** |  |
| **2.** | From a seated position, bend your trunk to the right side and then to the left side, then, return to an upright. | **4** |  |
| **3.** | From a seated position, turn your trunk to the right side and then left side facing your back side. Be sure to keep your spine straight and don’t twist to the point of pain. | **4** | **The part about “facing your back side” is not clear. I don’t understand what is expected here. The trunk rotation to left and right is clear** |

**A. Training for Trunk Strength**

|  | **Activity** | **Progression/Adaptation** | **Rating** | **Comments** |
| --- | --- | --- | --- | --- |
|  |  |  |  |  |
| **1.** | In a upright sitting position bend forward while placing your hands on your knees, and return to upright sitting position by focusing on using your core to pull yourself up | *Progression*: increase the number of repetitions and speed. | **2**  **Will be 4 if done without hands on knees** | **Increasing speed for a ‘gravity dependent’ activity reduces the demand on the eccentric control. Is this what you want for progression of this activity – please think about all activities in relation to consequences of increased speed against key muscle activity**  **Hands on knees may ‘take away’ from potential to activate some trunk muscles if patient happens to absorb effect of gravitation force pull through their arms** |
| **2.** | Clasp your hands together, and then punch forward while keeping your arms parallel to the floor. Use your back muscles to come back up. | *Progression*: increase the number of repetitions.  *Adaptation*: try punching to either sides. | **4** | **I don’t understand this activity. Initially I thought it is about punching forward and the parallel to the floor even confirmed my understanding. I then got confused by the ‘use of back muscles to come up – coming up from what position??**  **My rating is based on understanding that they remain in an upright seated position while punching** |
| **3.** | From a comfortable lying position, hug your knees into your chest. Then, hold on to left leg with your left arm and then extend your right leg onto the floor. This is your starting position. From your starting position, bring you right leg back into your chest. Try not to use your leg muscles to achieve this movement. Focus specifically on engaging your core and using your core muscles to lift your leg up. Once your leg is back in your chest, give your core muscles a good squeeze, and then release your leg back down. | *Progression*: increase the number of repetitions | **2** | **A Picture is required and also have to use lay terms (extension, core….not typical every day terms)**  **I don’t understand: “Try not to use your leg muscles to achieve this movement” – how are they supposed to bring the leg up without using leg muscles. Find another instruction that will force them to also use core while bringing leg up. You don’t want to find yourself in a situation where they use more of momentum than core.**  **Clarification required for lay person: ..”give your core muscles a good squeeze” – what does this mean – what exactly must they do to make sure they get the tight squeeze of ‘core’?** |
| **4.** | While lying on your back, lift your legs up and bend your knees at a 90 degree angle. Your shins should be parallel to the floor and your thighs should be perpendicular. Your core should be fully engaged. This is your starting position, which is also referred to as tabletop position. From tabletop position, bring your left leg down and gently tap the floor with your left foot. Then, bring your leg back up by using your core muscles. Maintain a 90 degree bend in your knee the entire time. Repeat on the other leg, all while keeping your core as tight as possible. This completes one set. | *Progression*: increase the number of repetitions | **2** |  |

**ACTIVITIES FOR BALANCE**

**Warm up**

|  | **Training** | **Rating** | **Comments** |
| --- | --- | --- | --- |
| **1.** | Hold onto the chair or counter, and raise yourself up onto your tiptoes, keeping your knees straight and holding your upper body tall. Lower yourself back to the floor slowly, and repeat. | **4** | **Rating is based on assumption is that this is for a person preparing for standing balance activities** |
| **2.** | Stand with your feet flat on the floor and your arms at your sides. Raise yourself to tiptoe, keeping your upper body and knees straight. Slowly lower and repeat. Without support. | **1** | **The ‘without support; at the end is confusing. Surely if they can do this activity that includes raising to tiptoe without support as a warm up activity, they have very good balance** |

**Trainings**

**B. Training for Balance**

|  | **Activity** | **Progression/Adaptation** | **Rating** | **Comments** |
| --- | --- | --- | --- | --- |
| **1.** | Place tape on the floor in a straight line. Step sideways to cross the line, crossing one leg across the front of the other leg. Reverse the motion to return to the starting point, this time crossing a leg behind. With support. | *Progression*: increase speed and the number of repetitions | 2 | Appropriate for advanced rehabilitation, especially the crossing of legs walking backwards- this does not translate into everyday activities. Crossing of legs is usually done when walking forward. |
| **2.** | Perform the side step, crossing your legs across each other as you move sideways across a straight line, but without holding on. Go slowly to avoid a fall, and be ready to grab a hold of something if you lose your balance. | *Progression*: increase speed and the number of repetitions | 3 |  |
| **3.** | Using the straight tape line for side stepping, walk forward, placing the heel of your foot directly in front of the toe of your other foot as you walk. Continue to the end of the tape, turn, and repeat by returning to the starting point | *Progression*: increase speed and the number of repetitions | 3 |  |
| **4.** | Place your back against a wall, standing tall. Slowly lower into a squatting position, holding on with one hand if needed or not holding on at all. Move up to a standing position and repeat. | *Progression*: increase speed and the number of repetitions | 1 | Leaning against any form of support structure takes away from the balance reeducation component. This is just a strengthening exercise for the lower limbs which will translate into good balance - |
| **5.** | Place both feet flat on the floor. Slowly lift one leg until you are balanced on the other leg. Hold for a count of 10, and slowly lower it back down. Alternate legs and repeat. | *Progression*: increase number of repetitions and count  *Adaptation:* stretch out both hands to the side | 3 |  |
| **6.** | In a room that is free from obstacles, walk backwards slowly. Try to avoid looking where you are going, but use your sense of balance and slow movements to avoid a fall. At first, perform this exercise with something close by to hold onto like a wall or countertop until you gain confidence in your abilities. | *Progression*: increase speed and the number of repetitions | 1 | Backwards walking in home environment may not be easy to adhere to because obstacle free room may not be easy to find in most homes. It means they may have to do activity outdoors and hopefully it won’t be rough terrain. Rather focus more on forward walking with obstacles |
| **7.** | Slowly pass a slightly heavy object from hand to hand as you circle it around your body. Start by circling the body in a clockwise motion. Then, repeat in a counter-clockwise motion. Perform this exercise while standing. | *Progression*: increase speed and the number of repetitions | 1 | I don’t understand this exercise – the part about “as you circle it around your body’  Passing slightly heavy object from hand to hand would mean that the patient has very good upper limb function  It will be better to do an activity that requires them to just hold the ‘heavy object and move the arms instead of moving object from one hand to the other…  Think about – main challenge here is that I don’t even understand the second component of this activity |

**ACTIVITIES FOR LOWER EXTREMITY**

**Warm up**

|  | **Training** | **Rating** | **Comments** |
| --- | --- | --- | --- |
| **1.** | From a seated position, extend the unaffected leg until it is parallel to the floor. Avoid locking your knee. Then, slowly bring your foot back down to the floor.   - Repeat with the affected leg, alternating back and forth between legs for a total of 20 repetitions (10 on each leg). | **2** | **I assume that extend will be replaced with straighten…..usual English terms** |
| **2.** | From a seated position, extend the unaffected leg until it is parallel to the floor. Avoid locking your knee. Then, slowly bring your foot back down to the floor. | **1** | **What is the difference between this and number 1? It is not relevant because this is the same as number one if I understand these instructions correctly** |
| **3.** | From a seated position, lift your affected leg up into your chest, trying your best to maintain controlled movement. | **1** | **They should lift unaffected in order to balance while using affected leg for support** |
| **4.** | Then place your foot back down onto the floor. Repeat on the other leg, alternating back and forth for a total of 10 repetitions. | **4** |  |
| **5.** | Start with your affected leg still crossed over your other leg. Then, flex your foot back towards your shin – a movement known as dorsiflexion. If you cannot do this, use your hand to assist your foot through the movement. Repeat 10 times. | **1** | **Why do you say ‘still crossed’ – when was it crossed**  **Not clear what this is supposed to achieve – if they can’t dorsiflex, asking them to lean forward to assist foot is even more challenging to balance – I don’t understand why it is presented as something they should do if they struggle. If they can’t dorsiflex because of weak e.g. tibialis anterior) then that is purely a lower limb strength problem and asking them to lean forward to do passive or active assisted dorsiflexion in sitting will challenge their balance far more the original step – that is why I don’t understand what the goal of this next step is in relation to balance, not ankle movement** |

**Trainings**

1. **Trainings for Transfers from sit to stand**

|  | **Activity** | **Progression/Adaptation** | **Rating** | **Comments** |
| --- | --- | --- | --- | --- |
| **1.** | In sitting, lift up the affected leg and place foot to marks on the ground aiming for control and accuracy | *Progression*: Increase the speed and the number of repetitions | **1** |  |
| **2.** | Stand up from a sitting position on the edge of bed with the support of the unaffected hand | *Progression*: Increase the speed and the number of repetitions  *Adaptation*: do the task without support | **4** |  |
| **3.** | Sit-to-stand from chair by placing the affected foot behind | *Progression:* Reduce the height of the chair, hand support, and increase speed  *Adaptation*: Place the feet in self-selected position | **4** | **Indicate that they reduction of seating level height and not necessarily chair but any surface also including compliant surface such as bed and sofas/couch and doing it with and later without use of upper limb for support** |
| **4.** | Sit-to-stand from a low chair by placing the affected foot behind | *Progression:* Increase the repetitions and distance between the legs with unaffected leg out in front  *Adaptation:* decrease the height of the chair | **1** | **This is covered in number 3 within progression** |

**D. Training for Maintaining Standing Position**

|  | **Activity** | **Progression/Adaptation** | **Rating** | **Comments** |
| --- | --- | --- | --- | --- |
| **1.** | In standing position lift the affected leg sideways with support nearby | *Progression:* Increase the repetitions and speed, do the task with unaffected leg  *Adaptation:* place the leg on a higher surface e.g. a piece of block | **4** |  |
| **2.** | Rise and lower yourself from a high surface (e.g. a block) with support nearby | *Progression:* Increase the repetitions and speed, do the task starting with different leg  *Adaptation:* increase the height of the surface |  | **The rise and lower is not clear – is this heel raises of simulation of sit to stand – please clarify. I am not able to rate as I don’t understand the activity** |
| **3.** | While standing on the unaffected leg place the affected leg on a bottle (or any hard cylindrical object) then roll forwards and backwards with support | *Progression:* Increase the repetitions and speed | **2** | **This is beneficial but what is needed is more of ability to carry weight through affected leg while moving unaffected leg – use of antigravity muscles rather than open kinematic chain activity for affected leg unless this is preparation for swing phase of gait** |
| **4.** |  |  |  | **Is this supposed to be number 3 but standing on affected leg?? check** |

**E. Training for Reaching in Standing**

|  | **Activity** | **Progression/Adaptation** | **Rating** | **Comments** |
| --- | --- | --- | --- | --- |
| **1.** | While in standing position reach an object in front of you with the affected hand | *Progression*: Increase speed and the distance where the object is and reduce the hand support  *Adaptation*: Perform the activity with the paretic limb on a step | **3** | **Reduction of hand support? When was hand support encouraged – are they not supposed to do it without hand support from the onset?**  **What is the primary purpose of activities in this category – are they for upper limb function of for a combination of standing balance and upper limb function. If a combination some activities will have to focus primarily on balance and other on upper limb function** |
| **2.** | While in standing position raise your heel and touch an object above you with the affected hand | *Progression*: Increase speed and the height where the object is and reduce the hand support  *Adaptation*: Perform the activity with the paretic limb on a step | **2** |  |
| **3.** | Stand and reach for objects placed in varying positions and heights, such as low stool, high shelf, to the side | *Progression*: Increase speed and the height where the object is and reduce the hand support  *Adaptation*: Perform the activity with the paretic limb on a step | **2** | **Performing with paretic limb on step – not clear**  **If activities within this category are to improve dynamic standing balance, emphasis should be on reaching towards affected side and increasing distance of reach. Can also reach with unaffected side but still towards affected side to increase weight bearing through affected leg.**  **I don’t see this being emphasized in this section (E) and this makes me wonder whether the focus is on UL or balance. UL has been covered a lot in the other document. There is a need to focus more on balance while taking into consideration** |

**F. Training for Stepping and Walking**

|  | **Activity** | **Progression/Adaptation** | **Rating** | **Comments** |
| --- | --- | --- | --- | --- |
| **1.** | In standing, lift up the affected leg and place foot forward to marks on the ground aiming for control and accuracy | *Progression*: Increase the speed and the number of repetitions | **4** | **More emphasis for all activities in this section should be on minimizing use of upper limb for support** |
| **2.** | Take Step forward to a mark on the floor in front | *Progression*: Increase the speed and the number of repetitions  *Adaptation:* Lead with affected foot, then lead with unaffected foot | **4** |  |
| **3.** | Step forward onto a step with the paretic limb | *Progression*: Increase the speed and the number of repetitions  *Adaptation:* Lead with affected foot, then lead with unaffected foot | **4** |  |
| **4.** | Step up onto a step, starting with the affected leg, and step down, starting with the non-affected leg | *Progression*: Increase the height of the step and speed and reduce the hand support  *Adaptation*: Start with the unaffected limb when stepping up and down the step | **4** |  |
| **5.** | Walk over-ground stepping on marked points | *Progression*: Increase the speed and the number of repetitions  *Adaptation*: walk outdoors and be talking without looking at the ground | **4** |  |
