# Supplementary material for: Self-rehabilitation strategy for rural community-dwelling stroke survivors in a lower-middle income country: a modified Delphi study
Source: PLoS One. 2025 Feb 25;20(2):e0303658. doi: 10.1371/journal.pone.0303658 (PMC11856556; doi:10.1371/journal.pone.0303658)
Supplement: S6 File — (ZIP) [file pone.0303658.s006.zip › S6 Delphi 1 responses/P3.docx]

**TASK-SPECIFIC SELF-REHABILITATION TRAINING (TASSRET) FOR COMMUNITY-DWELLING STROKE SURVIVORS**

**ACTIVITIES FOR UPPER EXTREMITY**

**Warm up**

|  | **Training** | **Rating** | **Comments** |
| --- | --- | --- | --- |
| **1.** | In a seated position lace your fingers together and then, make large circular movements. You can use your non-affected arm to guide your affected arm. Make 10 slow controlled circles. | **4** | **This is a very good exercise to start with because the interlacing of fingers creates/stimulates an awareness of the affected arm. Perhaps it needs stated more directly whether the circles involve only wrist or full shoulder movement?** |
| **2.** | Start with your elbow on a table with your arm bent at 90 degrees. Then, curl your arm up just a little, and then release it back down just a little. Slowly repeat 10 times. | **4** | **Suggest adding “curl your fist and forearm towards your body”** |
| **3.** | From a seated position, gently prop yourself up on your affected arm about one foot away from your body. Then gently lean into it. You should feel a mild stretch on your affected side.   - If it feels good, hold the stretch for 10 seconds, and then return to center. Repeat on the other side for a total of 3 sets. - If it doesn’t feel good, stop the stretch immediately. | **3** | **I get the idea but I think it needs to be made a little clearer.** |
| **4.** | Place your elbow on a table, and then use your unaffected hand to stretch your affected hand at the wrist. Stretch backward, and then stretch forward. Perform this movement slowly for a total of 5 repetitions. | **4** | **Very important** |
| **5.** | Place your affected hand on the table with your palm down. Then, use your non-affected hand to slide your hand to the left and then to the right. Focus on initiating the movement solely from your wrist. Repeat slowly for a total of 10 repetitions. | **4** | **Very important** |
|  |  |  |  |

**Trainings**

**A. Trainings for reaching**

|  | **Activity** | **Progression/Adaptation** | **Rating** | **Comment** |
| --- | --- | --- | --- | --- |
|  | ***Where the use of an object is involved, the initial position of the object should be about 15cm (0.15m) from you in all training, and start with 200 repetitions of each task in a session.*** | ***After every week increase the distance of the object by 7.5cm (0.075m) and the number of repetitions by 10% (20 repetitions).*** | **4** |  |
| **1.** | Stretch out the affected arm to reach and touch an object on a table directly in front of you | *Progression*: Increase the speed and the number of repetitions  *Adaptation*: Increase the distance of the object from you | 4 |  |
| **2.** | Stretch out the affected arm to reach and touch an object on a table placed on the affected side | *Progression*: Increase the speed and the number of repetitions  *Adaptation*: Increase the distance of the object from you | 4 | Very important to stimulate neural pathways on the affected side. |
| **3.** | Stretch out the affected arm to reach and touch an object on a table placed on the unaffected side | *Progression*: Increase the speed and the number of repetitions  *Adaptation*: Increase the distance of the object from you | 4 | Very important |
| **4.** | Stretch out the affected arm to reach and touch an object on the floor | *Progression*: Increase the speed and the number of repetitions  *Adaptation*: Increase the distance of the object from you | 4 |  |
| **5**. | Lift up the affected arm to reach and touch an object hanged above your head | *Progression*: Increase the speed and the number of repetitions  *Adaptation*: Increase the distance of the object from you | 4 | Perhaps clarify… e.g. don’t use your unaffected arm to lift. |
| **6.** | With the affected hand touch the unaffected shoulder and return your hand to the initial position | *Progression*: Increase the number of repetitions and move the hand from the shoulder across the arm and forearm to the back of the hand | 4 | Midline crossing is really important. |
| **7.** | Using the affected hand touch your head and return your hand to the initial position | *Progression*: Increase the speed and the number of repetitions and move the hand towards the upper back | 4 |  |
| **8.** | With both hands touch your shoulders at once | *Progression*: Increase the speed and the number of repetitions | 4 | Very important. |
|  |  |  |  |  |

**B. Training for grasp/Grip**

|  | **Activity** | **Progression/Adaptation** | **Rating** | **Comment** |
| --- | --- | --- | --- | --- |
| **1.** | Place hand around object, try and squeeze object, then lift your fingers/thumb away from the object | *Progression*: Increase the speed and the number of repetitions  *Adaptation*: use different size and shape of the objects | 4 | Squeeze for how long? |
| **2.** | Place hand around object, try and squeeze object, lift it up from the table, then drop it back and lift your fingers/thumb away from the object | *Progression*: Increase the speed and the number of repetitions  *Adaptation*: use different size and shape of the objects | 4 |  |
| **3.** | Lift up the affected arm to reach an object hanged above your head, then hold the object between fingers and thumb, release it and put down your arm | *Progression*: Increase the speed and the number of repetitions  *Adaptation*: use different size and shape of the objects | 4 | Perhaps use raise not lift. |
| **4.** | Stretch out the affected arm to reach an object on the floor, then hold the object between fingers and thumb, release it and return to the starting position | *Progression*: Increase the speed and the number of repetitions  *Adaptation*: use different size and shape of the objects | 3 |  |

**C. Training for moving objects**

|  | **Activity** | **Progression/Adaptation** | **Rating** | **Comment** |
| --- | --- | --- | --- | --- |
| **1.** | Place hand around an object, hold it between fingers and thumb, lift it up from the table and transfer it to another position then pick it again and return it to its initial position | *Progression*: Increase the speed, number of repetitions and the distance between the two positions  *Adaptation*: use different size and shape of the objects | 4 | Another position where? |
| **2.** | Lift an object from a higher level to a lower level and vice versa | *Progression*: Increase the speed, number of repetitions and the distance between the two positions  *Adaptation*: use different size and shape of the objects | 4 |  |
| **3.** | Take lid of a bottle or a jar and return it in place | *Progression*: Increase the speed and the number of repetitions  *Adaptation*: use different size and shape of bottles and jars | 4 |  |
| **4.** | Open a food bowl, place the cover down and then replace it back | *Progression*: Increase the speed and the number of repetitions  *Adaptation*: use different size and shape of food bowl | 4 |  |
| **5.** | Using both hands pick up a plate and transfer it to another position | *Progression*: Increase the speed and the number of repetitions  *Adaptation*: use different size and shape of plates, practice different positions in terms of height level | 4 |  |
| **6.** | With the affected hand take a cup to your mouth | *Progression*: Increase the speed and the number of repetitions and use cups with handle  *Adaptation*: : use different size and shape of cups | 4 |  |
|  |  |  |  |  |

**D. Training for object manipulation**

|  | **Activity** | **Progression/Adaptation** | **Rating** | **Comment** |
| --- | --- | --- | --- | --- |
| **1.** | Use both hands to fold and unfold a piece of cloth | *Progression*: Increase the speed and the number of repetitions  *Adaptation*: use different kind of sheets including piece of cloth, bed sheets etc. | 4 | Important for coordination. |
| **2.** | Open covered pots of different sizes and transfer any powdered substance to a cup with a spoon, then close the pot | *Progression*: Increase the speed and the number of repetitions  *Adaptation*: Increase the diameter of the spoon | 4 | Important for coordination. |
| **3.** | Open a box, pick up objects inside the box, and transfer them to a pot, then close the box | *Progression*: Increase the speed and the number of repetitions  *Adaptation*: Increase the size of the objects | 4 | Important for coordination and planning. |
| **4.** | Pick up coins and small stones on the table or from the floor, and put the stones in a pot and gather the coins | *Progression*: Increase both the speed and the number of coins and stones  *Adaptation*: use different sizes of stones | 4 | Important for fine motor coordination. |
| **5.** | Open a box with key, pick up objects inside the box, and transfer them to a pot, then lock the box | *Progression*: Increase the speed and the number of repetitions  *Adaptation*: Increase the thickness of the key and size of the objects | 4 |  |
| **6.** | Pick up and transfer jars, bottles, and cups of different sizes and weights located on a table or from the floor. Transfer the liquid contents from jars and bottles to cups | *Progression*: Increase the speed as well as the distance from the object to be reached  *Adaptation*: Reduce the volume of liquid in the jars and bottles | 4 |  |
| **7.** | Take money in and out of the pocket | *Progression*: Increase the speed and the number of repetitions  *Adaptation*: use different pockets on your clothe | 3 | Similar to 4. But functionally important. |
| **8.** | Use both hands to pick up cap and place it on your head | *Progression*: Increase the speed and the number of repetitions  *Adaptation*: use different types of caps | 3 | “place” – functionally important. |
| **9.** | Use both hands to tie wrapper around your body | *Progression*: Increase the speed and the number of repetitions  *Adaptation*: use other things like head scarf | 3 |  |
| **10.** | With affected hand pick nuts from a container and break it’s shell | *Progression*: Increase the number of repetitions  *Adaptation*: bring out the nut and place it in another container | 3 |  |

**E. Training for hand/fingers precision**

|  |
| --- |
|  |

|  | **Activity** | **Progression/Adaptation** | **Rating** | **Comment** |
| --- | --- | --- | --- | --- |
| **1.** | Touch the tip of each finger with the tip of the thumb | *Progression*: Increase the speed and the number of repetitions, also touch the proximal, middle and the distal phalanx of each finger  *Adaptation:* do it without looking at the hand | 4 |  |
| **2.** | Pick up coins or small stones on the table from a particular point to a marked point | *Progression*: Increase the speed and the number of repetitions  *Adaptation*: use different sizes of stones | 4 |  |
| **3.** | Use both hands to button and unbutton your shirt | *Progression*: Increase the speed and the number of repetitions  *Adaptation*: do the task without looking | 4 | Very functionally important. |
| **4.** | Use the affected hand to press numbers on a phone with the index finger | *Progression*: Increase the speed and the number of repetitions and use other fingers  *Adaptation*: type text | 4 |  |

**ACTIVITIES FOR THE TRUNK**

**Warm up**

|  | **Training** | **Rating** | **Comments** |
| --- | --- | --- | --- |
| **1.** | From a seated position, press your back against the back rest. | **4** | **Stimulating trunk awareness.** |
| **2.** | From a seated position, bend your trunk to the right side and then to the left side, then, return to an upright. | **4** |  |
| **3.** | From a seated position, turn your trunk to the right side and then left side facing your back side. Be sure to keep your spine straight and don’t twist to the point of pain. | **4** | **“turn/twist…”** |

**A. Training for Trunk Strength**

|  | **Activity** | **Progression/Adaptation** | **Rating** | **Comments** |
| --- | --- | --- | --- | --- |
|  |  |  |  |  |
| **1.** | In a upright sitting position bend forward while placing your hands on your knees, and return to upright sitting position by focusing on using your core to pull yourself up | *Progression*: increase the number of repetitions and speed. | **4** |  |
| **2.** | Clasp your hands together, and then punch forward while keeping your arms parallel to the floor. Use your back muscles to come back up. | *Progression*: increase the number of repetitions.  *Adaptation*: try punching to either sides. | **4** | **Perhaps clarify “come back up”** |
| **3.** | From a comfortable lying position, hug your knees into your chest. Then, hold on to left leg with your left arm and then extend your right leg onto the floor. This is your starting position. From your starting position, bring you right leg back into your chest. Try not to use your leg muscles to achieve this movement. Focus specifically on engaging your core and using your core muscles to lift your leg up. Once your leg is back in your chest, give your core muscles a good squeeze, and then release your leg back down. | *Progression*: increase the number of repetitions | **4** |  |
| **4.** | While lying on your back, lift your legs up and bend your knees at a 90 degree angle. Your shins should be parallel to the floor and your thighs should be perpendicular. Your core should be fully engaged. This is your starting position, which is also referred to as tabletop position. From tabletop position, bring your left leg down and gently tap the floor with your left foot. Then, bring your leg back up by using your core muscles. Maintain a 90 degree bend in your knee the entire time. Repeat on the other leg, all while keeping your core as tight as possible. This completes one set. | *Progression*: increase the number of repetitions | **4** |  |
|  |  |  |  |  |

**ACTIVITIES FOR BALANCE**

**Warm up**

|  | **Training** | **Rating** | **Comments** |
| --- | --- | --- | --- |
| **1.** | Hold onto the chair or counter, and raise yourself up onto your tiptoes, keeping your knees straight and holding your upper body tall. Lower yourself back to the floor slowly, and repeat. | **4** |  |
| **2.** | Stand with your feet flat on the floor and your arms at your sides. Raise yourself to tiptoe, keeping your upper body and knees straight. Slowly lower and repeat. Without support. | **4** |  |
|  |  |  |  |
|  |  |  |  |

**Trainings**

**B. Training for Balance**

|  | **Activity** | **Progression/Adaptation** | **Rating** | **Comments** |
| --- | --- | --- | --- | --- |
| **1.** | Place tape on the floor in a straight line. Step sideways to cross the line, crossing one leg across the front of the other leg. Reverse the motion to return to the starting point, this time crossing a leg behind. With support. | *Progression*: increase speed and the number of repetitions | 3 |  |
| **2.** | Perform the side step, crossing your legs across each other as you move sideways across a straight line, but without holding on. Go slowly to avoid a fall, and be ready to grab a hold of something if you lose your balance. | *Progression*: increase speed and the number of repetitions | 3 |  |
| **3.** | Using the straight tape line for side stepping, walk forward, placing the heel of your foot directly in front of the toe of your other foot as you walk. Continue to the end of the tape, turn, and repeat by returning to the starting point | *Progression*: increase speed and the number of repetitions | 4 |  |
| **4.** | Place your back against a wall, standing tall. Slowly lower into a squatting position, holding on with one hand if needed or not holding on at all. Move up to a standing position and repeat. | *Progression*: increase speed and the number of repetitions | 4 |  |
| **5.** | Place both feet flat on the floor. Slowly lift one leg until you are balanced on the other leg. Hold for a count of 10, and slowly lower it back down. Alternate legs and repeat. | *Progression*: increase number of repetitions and count  *Adaptation:* stretch out both hands to the side | 4 |  |
| **6.** | In a room that is free from obstacles, walk backwards slowly. Try to avoid looking where you are going, but use your sense of balance and slow movements to avoid a fall. At first, perform this exercise with something close by to hold onto like a wall or countertop until you gain confidence in your abilities. | *Progression*: increase speed and the number of repetitions | 3 |  |
| **7.** | Slowly pass a slightly heavy object from hand to hand as you circle it around your body. Start by circling the body in a clockwise motion. Then, repeat in a counter-clockwise motion. Perform this exercise while standing. | *Progression*: increase speed and the number of repetitions | 4 |  |
|  |  |  |  |  |

**ACTIVITIES FOR LOWER EXTREMITY**

**Warm up**

|  | **Training** | **Rating** | **Comments** |
| --- | --- | --- | --- |
| **1.** | From a seated position, extend the unaffected leg until it is parallel to the floor. Avoid locking your knee. Then, slowly bring your foot back down to the floor.   - Repeat with the affected leg, alternating back and forth between legs for a total of 20 repetitions (10 on each leg). | **4** |  |
| **2.** | From a seated position, extend the unaffected leg until it is parallel to the floor. Avoid locking your knee. Then, slowly bring your foot back down to the floor. | **4** |  |
| **3.** | From a seated position, lift your affected leg up into your chest, trying your best to maintain controlled movement. | **4** |  |
| **4.** | Then place your foot back down onto the floor. Repeat on the other leg, alternating back and forth for a total of 10 repetitions. | **4** | **Are 3 and 4 meant to be the same one?** |
| **5.** | Start with your affected leg still crossed over your other leg. Then, flex your foot back towards your shin – a movement known as dorsiflexion. If you cannot do this, use your hand to assist your foot through the movement. Repeat 10 times. | **4** |  |

**Trainings**

1. **Trainings for Transfers from sit to stand**

|  | **Activity** | **Progression/Adaptation** | **Rating** | **Comments** |
| --- | --- | --- | --- | --- |
| **1.** | In sitting, lift up the affected leg and place foot to marks on the ground aiming for control and accuracy | *Progression*: Increase the speed and the number of repetitions | **3** |  |
| **2.** | Stand up from a sitting position on the edge of bed with the support of the unaffected hand | *Progression*: Increase the speed and the number of repetitions  *Adaptation*: do the task without support | **4** |  |
| **3.** | Sit-to-stand from chair by placing the affected foot behind | *Progression:* Reduce the height of the chair, hand support, and increase speed  *Adaptation*: Place the feet in self-selected position | **4** |  |
| **4.** | Sit-to-stand from a low chair by placing the affected foot behind | *Progression:* Increase the repetitions and distance between the legs with unaffected leg out in front  *Adaptation:* decrease the height of the chair | **4** |  |

**D. Training for Maintaining Standing Position**

|  | **Activity** | **Progression/Adaptation** | **Rating** | **Comments** |
| --- | --- | --- | --- | --- |
| **1.** | In standing position lift the affected leg sideways with support nearby | *Progression:* Increase the repetitions and speed, do the task with unaffected leg  *Adaptation:* place the leg on a higher surface e.g. a piece of block | **4** |  |
| **2.** | Rise and lower yourself from a high surface (e.g. a block) with support nearby | *Progression:* Increase the repetitions and speed, do the task starting with different leg  *Adaptation:* increase the height of the surface | **4** | **Do you mean step up and step down?** |
| **3.** | While standing on the unaffected leg place the affected leg on a bottle (or any hard cylindrical object) then roll forwards and backwards with support | *Progression:* Increase the repetitions and speed | **4** |  |
| **4.** |  |  |  |  |

**E. Training for Reaching in Standing**

|  | **Activity** | **Progression/Adaptation** | **Rating** | **Comments** |
| --- | --- | --- | --- | --- |
| **1.** | While in standing position reach an object in front of you with the affected hand | *Progression*: Increase speed and the distance where the object is and reduce the hand support  *Adaptation*: Perform the activity with the paretic limb on a step | **4** |  |
| **2.** | While in standing position raise your heel and touch an object above you with the affected hand | *Progression*: Increase speed and the height where the object is and reduce the hand support  *Adaptation*: Perform the activity with the paretic limb on a step | **4** |  |
| **3.** | Stand and reach for objects placed in varying positions and heights, such as low stool, high shelf, to the side | *Progression*: Increase speed and the height where the object is and reduce the hand support  *Adaptation*: Perform the activity with the paretic limb on a step | **4** | **Very functionally important** |
|  |  |  |  |  |

**F. Training for Stepping and Walking**

|  | **Activity** | **Progression/Adaptation** | **Rating** | **Comments** |
| --- | --- | --- | --- | --- |
| **1.** | In standing, lift up the affected leg and place foot forward to marks on the ground aiming for control and accuracy | *Progression*: Increase the speed and the number of repetitions | **4** |  |
| **2.** | Take Step forward to a mark on the floor in front | *Progression*: Increase the speed and the number of repetitions  *Adaptation:* Lead with affected foot, then lead with unaffected foot | **4** |  |
| **3.** | Step forward onto a step with the paretic limb | *Progression*: Increase the speed and the number of repetitions  *Adaptation:* Lead with affected foot, then lead with unaffected foot | **4** |  |
| **4.** | Step up onto a step, starting with the affected leg, and step down, starting with the non-affected leg | *Progression*: Increase the height of the step and speed and reduce the hand support  *Adaptation*: Start with the unaffected limb when stepping up and down the step | **4** |  |
| **5.** | Walk over-ground stepping on marked points | *Progression*: Increase the speed and the number of repetitions  *Adaptation*: walk outdoors and be talking without looking at the ground | **4** |  |
