# Supplementary material for: Self-rehabilitation strategy for rural community-dwelling stroke survivors in a lower-middle income country: a modified Delphi study
Source: PLoS One. 2025 Feb 25;20(2):e0303658. doi: 10.1371/journal.pone.0303658 (PMC11856556; doi:10.1371/journal.pone.0303658)
Supplement: S7 File — (ZIP) [file pone.0303658.s007.zip › S7 Delphi 2 responses/P1.docx]

**TASK-SPECIFIC SELF-REHABILITATION TRAINING (TASSRET) FOR COMMUNITY-DWELLING STROKE SURVIVORS**

**ACTIVITIES FOR UPPER EXTREMITY**

**Warm up**

1. In an upright seated position, lace your fingers with your upper limb straight forward and make large circular movements in the horizontal plane involving full shoulder movements. You can use your non-affected arm to guide the affected arm. Make 10 slow controlled circles.
2. In an upright seated position, place your elbow on a table with your forearm bent at 90 degrees at the elbow joint and supinated, and the arm attached to the trunk. Then curl your hand and forearm towards your shoulder, and then release it back down. Slowly repeat 10 times.

**A: Trainings for reaching**

|  | **Training** | **Progression/Adaptation** | **Rating** | **Comment** |
| --- | --- | --- | --- | --- |
| 1 | In an upright seated position, stretch out the affected arm to reach and touch an object on a table directly in front of you, make sure you move the upper limb and not compensate a lot with trunk movement. | Do same task with the object placed at about 15cm from its original position on affected side and then on the unaffected side. Use both hands do to the same tasks. | 4 |  |
| 2 | Using the affected hand, reach and touch an object hanged at a height of about 15cm in front of you in a visible position. | Use both hands to do the same task. | 3 |  |
| 3 | Using the affected hand touch your head and return your hand to the initial position (see starting position above under instruction). |  | 1 |  |
| 4 | With both hands touch your shoulders at once (each hand to the opposite side shoulder) crossing the forearms. Then return your hand to the initial position (see starting position above under instruction) |  | 1 |  |

**B**: **Training for grasp/Grip and moving objects**

| 1 | Place the affected hand around an object, lift it up to about 15cm from the table, then place it back and release the object from your fingers/thumb and return to the starting position. |  | **4** |  |
| --- | --- | --- | --- | --- |
| 2 | Place the affected hand around an object, hold it between fingers and thumb, lift it up from the table and move it to about 30cm towards the affected side. Then pick it again and return it to its initial position. Do the same towards the unaffected side. |  | 4 |  |
| 3 | Using the affected hand lift an object (e g water bottle) from the table and place it on the floor by the affected side and return to starting position. Then pick the object and place it on the table in its initial position. |  | 3 |  |
| 4 | Take lid of a bottle or a jar and return it in place. | Progress from bigger to smaller lid. | 4 |  |
| 5 | Open the cover of a local eating bowl, place the cover down on the table and then place it back |  | 4 | 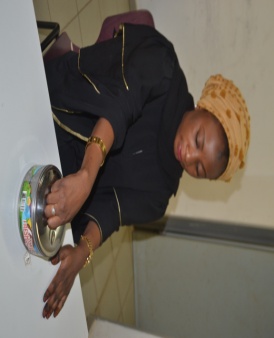4&5 can be combined? |
| 6 | While holding a plate with the unaffected hand, pick up a piece of cloth with the affected hand and clean the plate. |  | 4 |  |
| 7 | With the affected hand take a cup to your mouth (see instruction in the beginning of this section about positioning and using an object). |  | 4 |  |
| 8 | Raise up the affected arm to reach an object hanged above your head in the front in a visible position, then grab the object, release it and put down your arm. |  | 3 |  |

**D: Training for object manipulation**

| 1 | Use both hands to fold and unfold a piece of cloth placed on a table. |  | 4 |  |
| --- | --- | --- | --- | --- |
| 2 | Use the affected hand to open covered containers (containing any form of powdered substance, e.g. jars of sugar etc.) of different sizes and transfer the powdered substance with a spoon into a cup, then close the pot. |  | 4 |  |
| 3 | Use the affected hand to open a box (containing some objects), and pick up the objects and transfer them to a pot, then close the box. |  | 4 |  |
| 4 | Using the affected hand pick up coins and small stones on the table, put the stones in a pot placed on the table and collate the coins together. | Do same with the pot placed on the floor. | 4 |  |
| 5 | Lock and unlock a pad locks of different sizes (with key) | Start with bigger padlocks then progress with smaller. | 4 |  |
| 6 | Pour liquid contents from jars and bottles to cups. Involve both hands and do normal movement as much as possible. | Start with bigger jars and bottles then progress with smaller. | 4 |  |
| 7 | Take money (notes) in and out of the pocket using the affected hand | Do the training using different pockets on your cloth. | 1 |  |
| 8 | Use the affected hand to pick up cap and place it on your head, then use both hands to adjust it well. For women use both hands to tie a head tie on your head. |  | 4 | 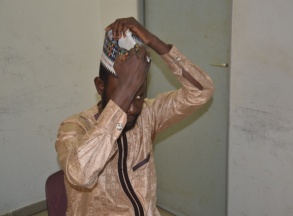 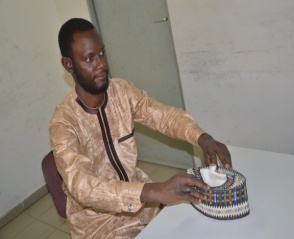 |
| 9 | Use both hands to tie wrapper around your body (for women). |  | 2 |  |
| 10 | While seated on a stool with a bucket containing some water, use both hands and immerse clothes in to the bucket of water and remove it, then squeeze the water out of the cloth by wringing it. |  | 4 |  |

**E: Training for hand/fingers precision**

| **1** | Use both hands to button and unbutton your shirt. |  | **4** |  |
| --- | --- | --- | --- | --- |
| **2** | Use the affected hand to press numbers and/or type texts on a mobile phone. |  | **4** |  |
| **3** | Pick a box of matches with the unaffected, then use the affected hand to push it open and pick a match stick out of the box. | Scratch the stick against the box to start a fire (necessary precaution should be taken to avoid fire outbreak). | **4** |  |
| **4** | With affected hand pick peanuts from a container use both hands to break its shell. (make sure you involve the affected hand as much as possible |  | **3** |  |

**ACTIVITIES FOR THE TRUNK**

**Warm up**

1. From the upright sitting position, rotate your trunk to the right, then return to the midline and rotate to the left (do it slowly).

**A: Training for Trunk Strength**

| 1 | From the upright sitting position bend your trunk forward while extending the affected hand to reach an object place on a table at a distance of about 60cm (initially), and come back to the upright sitting position (avoid using the arms to assist in either bending or coming back to the upright position as much as possible). |  | 3 |  |
| --- | --- | --- | --- | --- |
| 2 | From a seated position on a chair with back rest, press your back against the back rest to approximate upright sitting. Stay in that position for approximately 3 minutes. |  | 2 |  |
| 3 | In upright sitting position bend your trunk forward while stretching out the affected hand attempting to touch an object placed on the ground at about 5cm away from the affected leg, and return to upright sitting position by focusing on using your core to pull yourself up. |  | 2 |  |
| 4 | While lying on your back, lift your legs up and bend your knees at a 90 degree angle. Your shins should be parallel to the floor and your thighs should be perpendicular. Your core should be fully engaged. In this position wear trousers. Then go back to the starting position. |  | 3 |  |

**ACTIVITIES FOR LOWER EXTREMITY AND BALANCE**

**Warm up**

1. In a standing position, hold onto the chair or counter, and raise yourself up onto your tiptoes, keeping your knees straight and holding your upper body tall. Lower yourself back to the floor slowly, and repeat.

**A: Trainings for Transfers from sit to stand**

| 1 | Stand up from a sitting position on the edge of bed with the support of the unaffected hand. |  | 2 |  |
| --- | --- | --- | --- | --- |
| 2 | From an upright sitting position (on a chair) stand up by placing the unaffected foot forward until you stand upright. Stay for about 30 seconds and sit back. | Use shorter stool. | 3 |  |

**B: Trainings for maintaining standing position and reaching in standing**

| 1 | While in standing position use the affected hand to reach an object placed at the level of your shoulder (initially) at about 30cm in front of you. | Place the object above the shoulder level. | 1 | Exercise in number 2 below more appropriate? |
| --- | --- | --- | --- | --- |
| 2 | In standing position reach for objects positioned at about 30cm away from you on the affected side, and at the level of your shoulder (initially). | Place the object above the shoulder level, also use the unaffected hand to reach the same object at the same position. | 3 |  |

**C: Trainings for stepping and walking**

| 1 | In standing position, take a step forward with the affected leg and place foot on marks on the ground at an interval of about 70 to 80cm (for males) and 60 to 70cm (for females) aiming for control and accuracy. |  | 3 |  |
| --- | --- | --- | --- | --- |
| 2 | Walk up and down a 10m course at a steady pace without support, and avoid looking down. (a family caregiver should be around to provide assistance when needed, also a chair should be available for resting when and if you feel tired in between training). |  | 3 |  |
| 3 | Step up and down a surface of about 5cm height (at the start). Start by stepping up with the affected leg, and stepping down with the non-affected leg. | Step up with non-affected first, then step down with the affected leg, and use less support. Use higher surfaces to a maximum of 25cm high. | 4 |  |
| 4 | Walk out of your house and sit on a chair outside the house for 15 minutes then walk back inside the house. (a family caregiver should be nearby for any possible required assistance) | Walk to a distance of about 50m from the door of your house. | 4 |  |
