# Supplementary material for: Self-rehabilitation strategy for rural community-dwelling stroke survivors in a lower-middle income country: a modified Delphi study
Source: PLoS One. 2025 Feb 25;20(2):e0303658. doi: 10.1371/journal.pone.0303658 (PMC11856556; doi:10.1371/journal.pone.0303658)
Supplement: S8 File — (ZIP) [file pone.0303658.s008.zip › S8 Delphi 3 responses/P2.docx]

**TASK-SPECIFIC SELF-REHABILITATION TRAINING (TASSRET) FOR COMMUNITY-DWELLING STROKE SURVIVORS: GUIDELINES MANUAL**

**Preamble**

This manual comprised of a series of Task-specific exercises that are intended as a guide to direct and assist stroke survivors to self-rehabilitate in their own homes. Task-specific Self-rehabilitation Training (TASSRET) is designed as a model of therapy delivery to community-dwelling stroke survivors. TASSRET employs the same key principles that are applied in any task-oriented rehabilitation intervention, and the model is aimed at promoting independence and maximizing functional outcomes following discharge from hospital.

The model comprises a set of task-oriented training (arranged in order of progression and adaptation) for each of the following body parts or target functional task:

- Upper extremity function
- Lower extremity function
- Trunk and
- Balance functions

Before starting some set of exercise training, there are pre-exercise activities that should be performed as warm ups.

**General Guidelines and Instructions**

This package is intended for stroke survivors with the following characteristics:

- Have more than 10-degree range of motion of the index finger metacarpophalangeal joint.
- Having a Motorcity Index score between 42 and 93 points for both upper and lower extremity;
- Have the ability to stand and take a step (with or without support);
- Have cognitive ability to follow commands, as indicated by a score of 0 to 1 on the Commands item of the National Institutes of Health Stroke Scale or Mini-Mental State Examination score of ≥24.

It is important to perform the training independently as much as possible, however, a caregiver or a family member should be around during the training session to provide assistance when and only where necessary.

Everyday household items available and appropriate and that would not cause harm to the patient should be used as much as possible in the training where applicable. Even though, the aim is to achieve a perfect movement pattern for tasks, however, these **Practices don’t necessarily have to be perfect!**

***Frequency*** – twice daily (1 hour per session), 3 days a week.

**ACTIVITIES FOR UPPER EXTREMITY**

***Instruction****:* ***Positioning for the activities in this section has the patient seated upright in a chair with a firm back and no armrests, and the trunk in contact with the back of the chair. The head should be in a neutral position, with feet in contact with the floor and the hands resting on the lap.***

***The subject sits close to a table, within 15cm (0.15m) distance from the anterior torso to the front edge of the table. Where the use of an object is involved, the initial position of the object should be about 30cm (0.3m) from the anterior torso in all training, and start with 10 repetitions of each task in a session. After every week increase the distance of the object by 7.5cm (0.075m) till you reach a maximum distance of 50cm (0.5m), and the number of repetitions be increased by 50% of the starting number of repetitions weekly (5 repetitions).***

***Objects to be used may include bottle of water and cups of different sizes etc*.**

**Warm up**

1. In an upright seated position, lace your fingers with your upper limb straight forward and make large circular movements in the horizontal plane involving full shoulder movements. You can use your non-affected arm to guide the affected arm. Make 10 slow controlled circles.
2. In an upright seated position, place your elbow on a table with your forearm bent at 90 degrees at the elbow joint and supinated, and the arm in contact with the trunk. Then curl your hand and forearm towards your shoulder, and then release it back down. Slowly repeat 10 times.
3. In an upright seated position, place your affected hand on the table with your palm down, then slide your hand to the left and then to the right at the wrist joint. Focus on initiating the movement solely from your wrist. You may use the unaffected hand to assist in moving the affected hand.

**A: Trainings for reaching**

|  | **Training** | **Progression/Adaptation** | **Final remarks** |
| --- | --- | --- | --- |
| 1 | In an upright seated position, stretch out the affected arm to reach and touch an object on a table directly in front of you, make sure you move the upper limb and not compensate a lot with trunk movement. | Do same task with the object placed at about 15cm from its original position on affected side and then on the unaffected side. Use both hands do to the same tasks. | 4 |
| 2 | Using the affected hand, reach and touch an object hanged at a height of about 15cm (from the surface of a table) in front of you in a visible position. | Use both hands to do the same task. | 4 |
| 3 | Using the affected hand touch your head and return your hand to the initial position (see starting position above under instruction). |  | 3 |
| 4 | With both hands touch your shoulders at once (each hand to the opposite side shoulder) crossing the forearms. Then return your hand to the initial position (see starting position above under instruction) |  | 3 |

**B**: **Training for grasp/Grip and moving objects**

| 5 | Place the affected hand around a cup, lift it up to about 15cm from the table, then place it back and release the object from your fingers/thumb and return to the starting position. |  | 4 |
| --- | --- | --- | --- |
| 6 | Place the affected hand around a bottle of water, hold it between fingers and thumb, lift it up from the table and move it sideways to about 30cm on the affected side and place it down. Then pick it again and return it to its initial position. Do the same towards the unaffected side. |  | 4 |
| 7 | Using the affected hand lift an object (e g water bottle) from the table and place it on the floor on the affected side and return to starting position. Then pick the object and place it on the table in its initial position. |  | 4 |
| 8 | Take off the lid of a bottle or a jar by unscrewing the cap and return it in place. | Progress from bigger to smaller lid. | 4 |
| 9 | Open the cover of an eating bowl, place the cover down on the table and then replace it back on the bowl. |  | 4 |
| 10 | While holding a plate with the unaffected hand, pick up a piece of cloth (placed on a table in front of you) with the affected hand and clean the plate. |  | 4 |
| 11 | With the affected hand take a cup of clean water to your mouth, and take a sip (see instruction in the beginning of this section about positioning and using an object). |  | 4 |
| 12 | Raise up the affected arm to reach an object hanged above and in front of your head in a visible position, then grab the object, hold for 2/3 seconds then release it and put down your arm. |  | 4 |

**D: Training for object manipulation**

| 13 | Use both hands to fold and unfold a piece of cloth placed on a table. |  | 4 |
| --- | --- | --- | --- |
| 14 | Use the affected hand to open covered containers (containing any form of powdered substance, e.g. jars of sugar etc.) of different sizes and transfer the powdered substance with a spoon into a cup, then close the container. |  | 4 |
| 15 | Use the affected hand to open a box (containing some objects), and pick up the objects and transfer them to a pot, then close the box. |  | 3 |
| 16 | Mix two kind of nuts or grains in a container (e.g. pea nuts and tiger nuts), using the affected hand pick up the tiger nuts one by one and transfer them to another container. |  | 3 |
| 17 | With the elbows rested on the table, Unlock padlocks  With a keys. | Start with bigger padlocks then progress with smaller. | 4 |
| 18 | Pour liquid contents from a jar and/or bottle into cup(s). Involve both hands. | Start with bigger jars and bottles then progress with smaller. | 4 |
| 19 | With the elbows rested on the table, take money (notes) in and out of a wallet using the affected hand. |  | 4 |
| 20 | Use both hands to pick up cap and place it on your head, then use both hands to adjust it well. For women use both hands to tie a head tie on your head. |  | 4 |
| 21 | Use both hands to tie a wrapper (for women) and a towel around your waist. |  | 4 |
| 22 | While seated on a stool, with a bucket containing some water placed in front of you, use both hands and immerse a clothe in to the bucket of water and remove it, then squeeze the water out of the cloth by wringing it. |  | 4 |

**E: Training for hand/fingers precision**

| 23 | Use both hands to button and unbutton your shirt. |  | **4** |
| --- | --- | --- | --- |
| 24 | Use the affected hand to press numbers and/or type texts on a mobile phone (those with regular keyboards). |  | **4** |
| 25 | Pick up a box of matches with the unaffected hand, and then use the affected hand to push it open and pick a match out of the box. | Light a match (necessary precaution should be taken to avoid fire outbreak). | **4** |
| 26 | With affected hand pick peanuts from a container use both hands to break their shells. (make sure you involve the affected hand as much as possible |  | **4** |
| 27 | In a sitting position on a low stool, pour water from a kettle in to the affected hand and use it to wash your face |  | **4** |

**ACTIVITIES FOR THE TRUNK**

***Instruction:*** *A chair without arm rest and bed are needed in this round of activities. If you can’t fully complete exercise yet, then you can use your arm to support and assist yourself. If you feel any pain in your back, stop immediately.* ***Practice doesn’t have to be perfect!***

**Warm up**

- From the upright sitting position, bend to the right, come to upright, then bend to the left and come back to the upright (do it slowly).
- From the upright sitting position, rotate your trunk to the right, then return to the midline and rotate to the left (do it slowly).

**A: Training for Trunk Strength**

| 28 | From the upright sitting position bend your trunk forward while extending the affected hand to reach an object place on a table at a distance of about 60cm from the front edge of the table, and come back to the upright sitting position (avoid using the arms to assist in either bending or coming back to the upright position as much as possible). |  | 3 |
| --- | --- | --- | --- |
| 29 | From a seated position, press your back against the back rest of the chair to approximately upright sitting. Stay in that position for approximately 3 minutes. |  | 3 |
| 30 | In upright sitting position bend your trunk forward while stretching out the affected hand attempting to touch an object placed on the ground at about 5cm away from the affected leg, and return to upright sitting position by focusing on using your core to pull yourself up. |  | 3 |
| 31 | While lying on your back, lift your legs up and bend your knees at a 90 degree angle. Your shins should be parallel to the floor and your thighs should be perpendicular. Your core should be fully engaged. In this position wear a pair of shorts. Then go back to the starting position. |  | 4 |

**ACTIVITIES FOR LOWER EXTREMITY AND BALANCE**

***Instruction:*** *A care giver or any family member should be nearby and can provide minimal assistance when and where necessary. After practicing the basic level exercises for a while, you should be able to perform them without assistance. However, for safety, always have a counter or chair nearby to grab if you start to lose your balance. Avoid looking down while doing this round of training.* ***Practice doesn’t have to be perfect!***

**Warm up**

In a standing position, hold onto the chair or counter, and raise yourself up onto your tiptoes, keeping your knees straight and holding your upper body tall. Lower yourself back to the floor slowly, and repeat.

**A: Trainings for Transfers from sit to stand**

| 32 | Stand up from a sitting position on the edge of bed with the support of the unaffected hand. |  | 3 |
| --- | --- | --- | --- |
| 33 | From an upright sitting position (on a chair) stand up by placing the unaffected foot forward until you stand upright. Stay for about 30 seconds and sit back. | Use lower stool. | 4 |

**B: Trainings for maintaining standing position and reaching in standing**

| 34 | While in standing position use the affected hand to reach an object placed at the level of your shoulder (initially) at about 30cm in front of you. | Place the object above the shoulder level. | 4 |
| --- | --- | --- | --- |
| 35 | In standing position reach for objects positioned at about 30cm placed sideway to the affected side, and at the level of your shoulder (initially). | Place the object above the shoulder level, also use the unaffected hand to reach the same object at the same position. | 4 |

**C: Trainings for stepping and walking**

| 36 | In standing position, take a step forward with the affected leg and place foot on marks on the ground at an interval of about 70 to 80cm (for males) and 60 to 70cm (for females) aiming for control and accuracy. |  | 3 |
| --- | --- | --- | --- |
| 37 | Walk up and down a 10m course at a steady pace without support, and avoid looking down. (a family caregiver should be around to provide assistance when needed, also a chair should be available for resting when and if you feel tired in between training). |  | 4 |
| 38 | Step up and down a surface of about 5cm height (at the start). Start by stepping up with the affected leg, and stepping down with the non-affected leg. | Step up with non-affected first, then step down with the affected leg, and use less support. Use higher surfaces to a maximum of 25cm high. | 4 |
| 39 | Walk out of your house and sit on a chair outside the house for 15 minutes then walk back inside the house. (a family caregiver should be nearby for any possible required assistance) | Walk to a distance of about 50m from the door of your house. | 4 |
|  | **SCORE** |  | **1.00** |
