# Supplementary material for: Self-rehabilitation strategy for rural community-dwelling stroke survivors in a lower-middle income country: a modified Delphi study
Source: PLoS One. 2025 Feb 25;20(2):e0303658. doi: 10.1371/journal.pone.0303658 (PMC11856556; doi:10.1371/journal.pone.0303658)
Supplement: S10 File — (DOC) [file pone.0303658.s010.doc]

FIRST ROUND

NPAR TESTS
  /KENDALL=V4 V3 V5 V6 V7 V8 V9 V2 V10 V11 V12 V13 V14 V15 V16 V17 V18 V19 V20 V21 V22 V23 V24 V25
    V26 V27 V28 V29 V30 V31 V32 V33 V34 V35 V36 V37 V38 V39 V40 V41 V42 V43 V44 V45 V46 V47 V48 V49 V50
    V51 V52 V53 V54 V55 V56 V57 V58 V59 V60 V61 V62 V63 V64 V65 V66 V68 V69 V70 V71 V72 V73 V74 V75
  /STATISTICS DESCRIPTIVES
  /MISSING LISTWISE.


NPar Tests


Notes	
Output Created	07-OCT-2024 09:53:23	
Comments		
Input	Active Dataset	DataSet3	
	Filter	<none>	
	Weight	<none>	
	Split File	<none>	
	N of Rows in Working Data File	13	
Missing Value Handling	Definition of Missing	User-defined missing values are treated as missing.	
	Cases Used	Statistics for all tests are based on cases with no missing data for any variables used.	
Syntax	NPAR TESTS
  /KENDALL=V4 V3 V5 V6 V7 V8 V9 V2 V10 V11 V12 V13 V14 V15 V16 V17 V18 V19 V20 V21 V22 V23 V24 V25
    V26 V27 V28 V29 V30 V31 V32 V33 V34 V35 V36 V37 V38 V39 V40 V41 V42 V43 V44 V45 V46 V47 V48 V49 V50
    V51 V52 V53 V54 V55 V56 V57 V58 V59 V60 V61 V62 V63 V64 V65 V66 V68 V69 V70 V71 V72 V73 V74 V75
  /STATISTICS DESCRIPTIVES
  /MISSING LISTWISE.	
Resources	Processor Time	00:00:00.02	
	Elapsed Time	00:00:00.64	
	Number of Cases Alloweda	20164	

a. Based on availability of workspace memory.	


Descriptive Statistics	
	N	Mean	Std. Deviation	Minimum	Maximum	
3	7	2.71	.756	2	4	
2	7	3.00	.816	2	4	
4	7	3.00	.816	2	4	
5	7	3.71	.488	3	4	
6	7	3.71	.488	3	4	
7	7	3.71	.488	3	4	
8	7	3.71	.488	3	4	
1	7	2.57	1.134	1	4	
9	7	3.43	.787	2	4	
10	7	3.71	.488	3	4	
11	7	3.71	.488	3	4	
12	7	3.57	.535	3	4	
13	7	3.29	.488	3	4	
14	7	3.71	.488	3	4	
15	7	3.29	.488	3	4	
16	7	3.57	.535	3	4	
17	7	3.57	.535	3	4	
18	7	3.57	.535	3	4	
19	7	3.43	.787	2	4	
20	7	3.43	.787	2	4	
21	7	3.57	.535	3	4	
22	7	3.71	.488	3	4	
23	7	3.71	.488	3	4	
24	7	3.71	.488	3	4	
25	7	3.71	.488	3	4	
26	7	3.71	.488	3	4	
27	7	3.71	.488	3	4	
28	7	3.71	.488	3	4	
29	7	3.71	.488	3	4	
30	7	3.57	.535	3	4	
31	7	3.57	.535	3	4	
32	7	3.57	.535	3	4	
33	7	2.86	.690	2	4	
34	7	2.71	1.254	1	4	
35	7	3.57	.535	3	4	
36	7	3.29	.756	2	4	
37	7	3.57	.535	3	4	
38	7	2.86	1.345	1	4	
39	7	3.57	.535	3	4	
40	7	3.57	.787	2	4	
41	7	2.71	1.254	1	4	
42	7	3.29	.756	2	4	
43	7	2.86	1.345	1	4	
44	7	3.14	.690	2	4	
45	7	2.43	1.134	1	4	
46	7	3.14	1.215	1	4	
47	7	3.14	.690	2	4	
48	7	3.29	.756	2	4	
49	7	2.29	1.113	1	4	
50	7	3.29	.756	2	4	
51	7	2.71	1.254	1	4	
52	7	3.43	.535	3	4	
53	7	3.00	.816	2	4	
54	7	2.86	1.345	1	4	
55	7	2.86	1.345	1	4	
56	7	2.71	1.254	1	4	
57	7	2.71	1.254	1	4	
58	7	3.29	.488	3	4	
59	7	2.71	1.254	1	4	
60	7	2.71	1.254	1	4	
61	7	3.57	.535	3	4	
62	7	2.86	1.345	1	4	
63	7	3.71	.488	3	4	
64	7	3.57	.535	3	4	
65	7	3.57	.535	3	4	
67	7	3.71	.488	3	4	
68	7	3.71	.488	3	4	
69	7	3.71	.488	3	4	
70	7	3.71	.488	3	4	
71	7	3.71	.488	3	4	
72	7	3.71	.488	3	4	
73	7	3.71	.488	3	4	
74	7	3.57	.535	3	4	


Kendall's W Test


Ranks	
	Mean Rank	
3	24.86	
2	31.50	
4	31.00	
5	45.07	
6	45.07	
7	45.07	
8	45.07	
1	23.43	
9	38.93	
10	45.07	
11	45.07	
12	40.36	
13	32.29	
14	45.07	
15	32.29	
16	40.36	
17	39.86	
18	39.86	
19	34.64	
20	34.64	
21	40.36	
22	45.07	
23	45.07	
24	45.07	
25	45.07	
26	45.07	
27	45.07	
28	45.07	
29	45.07	
30	39.86	
31	39.86	
32	39.86	
33	25.79	
34	28.14	
35	39.86	
36	33.71	
37	39.86	
38	33.36	
39	40.36	
40	39.86	
41	28.64	
42	33.71	
43	33.36	
44	31.36	
45	18.21	
46	33.21	
47	24.71	
48	29.93	
49	16.79	
50	29.93	
51	28.64	
52	34.64	
53	31.50	
54	33.36	
55	33.36	
56	28.14	
57	28.14	
58	32.29	
59	28.14	
60	28.64	
61	39.86	
62	33.36	
63	45.07	
64	39.86	
65	39.86	
67	45.07	
68	45.07	
69	45.07	
70	45.07	
71	45.07	
72	45.07	
73	45.07	
74	39.86	


Test Statistics	
N	7	
Kendall's Wa	.252	
Chi-Square	127.076	
df	72	
Asymp. Sig.	.000	

a. Kendall's Coefficient of Concordance	

*Nonparametric Tests: Related Samples.
NPTESTS
  /RELATED TEST(V2 V3 V4 V5 V6 V7 V8 V9 V10 V11 V12 V13 V14 V15 V16 V17 V18 V19 V20 V21 V22 V23 V24 V25 V26 V27 V28 V29 V30 V31 V32 V33 V34 V35 V36 V37 V38 V39 V40 V41 V42 V43 V44 V45 V46 V47 V48 V49 V50 V51 V52 V53 V54 V55 V56 V57 V58 V59 V60 V61 V62 V63 V64 V65 V66 V68 V69 V70 V71 V72 V73 V74 V75) KENDALL(COMPARE=PAIRWISE)
  /MISSING SCOPE=ANALYSIS USERMISSING=EXCLUDE
  /CRITERIA ALPHA=0.05  CILEVEL=95.


Nonparametric Tests


Notes	
Output Created	07-OCT-2024 09:54:20	
Comments		
Input	Active Dataset	DataSet3	
	Filter	<none>	
	Weight	<none>	
	Split File	<none>	
	N of Rows in Working Data File	13	
Syntax	NPTESTS
  /RELATED TEST(V2 V3 V4 V5 V6 V7 V8 V9 V10 V11 V12 V13 V14 V15 V16 V17 V18 V19 V20 V21 V22 V23 V24 V25 V26 V27 V28 V29 V30 V31 V32 V33 V34 V35 V36 V37 V38 V39 V40 V41 V42 V43 V44 V45 V46 V47 V48 V49 V50 V51 V52 V53 V54 V55 V56 V57 V58 V59 V60 V61 V62 V63 V64 V65 V66 V68 V69 V70 V71 V72 V73 V74 V75) KENDALL(COMPARE=PAIRWISE)
  /MISSING SCOPE=ANALYSIS USERMISSING=EXCLUDE
  /CRITERIA ALPHA=0.05  CILEVEL=95.	
Resources	Processor Time	00:00:02.11	
	Elapsed Time	00:00:01.78	


SECOND ROUND

GET DATA /TYPE=XLSX
  /FILE='C:\Users\HP\OneDrive\Desktop\DELPHI\Delphi round 2.xlsx'
  /SHEET=name 'Sheet1'
  /CELLRANGE=full
  /READNAMES=on
  /ASSUMEDSTRWIDTH=32767.
EXECUTE.
DATASET NAME DataSet4 WINDOW=FRONT.
NPAR TESTS
  /KENDALL=V2 V3 V4 V5 V6 V7 V8 V9 V10 V11 V12 V13 V14 V15 V16 V17 V18 V19 V20 V21 V22 V23 V24 V25
    V26 V27 V28 V29 V30 V31 V32 V33 V34 V35 V36 V37 V38 V39 V40 V41 V42 V43
  /STATISTICS DESCRIPTIVES
  /MISSING LISTWISE.


NPar Tests


Notes	
Output Created	07-OCT-2024 10:09:12	
Comments		
Input	Active Dataset	DataSet4	
	Filter	<none>	
	Weight	<none>	
	Split File	<none>	
	N of Rows in Working Data File	13	
Missing Value Handling	Definition of Missing	User-defined missing values are treated as missing.	
	Cases Used	Statistics for all tests are based on cases with no missing data for any variables used.	
Syntax	NPAR TESTS
  /KENDALL=V2 V3 V4 V5 V6 V7 V8 V9 V10 V11 V12 V13 V14 V15 V16 V17 V18 V19 V20 V21 V22 V23 V24 V25
    V26 V27 V28 V29 V30 V31 V32 V33 V34 V35 V36 V37 V38 V39 V40 V41 V42 V43
  /STATISTICS DESCRIPTIVES
  /MISSING LISTWISE.	
Resources	Processor Time	00:00:00.03	
	Elapsed Time	00:00:00.03	
	Number of Cases Alloweda	33465	

a. Based on availability of workspace memory.	


[DataSet4] 


Descriptive Statistics	
	N	Mean	Std. Deviation	Minimum	Maximum	
1	10	3.70	.483	3	4	
2	10	3.30	.483	3	4	
3	10	3.90	.316	3	4	
4	10	3.10	.316	3	4	
5	10	3.30	1.252	1	4	
6	10	3.10	1.287	1	4	
7	10	4.00	.000	4	4	
8	10	3.90	.316	3	4	
9	10	3.70	.483	3	4	
10	10	4.00	.000	4	4	
11	10	3.90	.316	3	4	
12	10	4.00	.000	4	4	
13	10	3.90	.316	3	4	
14	10	3.60	.516	3	4	
15	10	4.00	.000	4	4	
16	10	4.00	.000	4	4	
17	10	3.90	.316	3	4	
18	10	4.00	.000	4	4	
19	10	4.00	.000	4	4	
20	10	4.00	.000	4	4	
21	10	3.40	1.075	1	4	
22	10	4.00	.000	4	4	
23	10	3.40	.843	2	4	
24	10	3.90	.316	3	4	
25	10	3.90	.316	3	4	
26	10	3.90	.316	3	4	
27	10	3.90	.316	3	4	
28	10	3.70	.483	3	4	
29	10	3.90	.316	3	4	
30	10	3.60	.516	3	4	
31	10	2.70	.483	2	3	
32	10	3.50	.850	2	4	
33	10	2.60	.699	2	4	
34	10	3.70	.483	3	4	
35	10	3.40	.843	2	4	
36	10	3.60	.516	3	4	
37	10	3.40	1.075	1	4	
38	10	3.60	.516	3	4	
39	10	3.60	.516	3	4	
40	10	3.70	.483	3	4	
41	10	4.00	.000	4	4	
42	10	4.00	.000	4	4	


Kendall's W Test


Ranks	
	Mean Rank	
1	20.50	
2	12.70	
3	24.60	
4	8.95	
5	18.95	
6	15.95	
7	26.70	
8	24.65	
9	21.25	
10	26.70	
11	24.65	
12	26.70	
13	24.65	
14	19.20	
15	26.70	
16	26.70	
17	24.65	
18	26.70	
19	26.70	
20	26.70	
21	19.25	
22	26.70	
23	17.65	
24	24.65	
25	24.65	
26	24.65	
27	24.65	
28	21.25	
29	24.70	
30	19.20	
31	4.20	
32	19.70	
33	7.80	
34	20.55	
35	17.65	
36	19.20	
37	19.20	
38	19.20	
39	19.20	
40	21.25	
41	26.70	
42	26.70	


Test Statistics	
N	10	
Kendall's Wa	.409	
Chi-Square	167.835	
df	41	
Asymp. Sig.	.000	

a. Kendall's Coefficient of Concordance	

*Nonparametric Tests: Related Samples.
NPTESTS
  /RELATED TEST(V2 V3 V4 V5 V6 V7 V8 V9 V10 V11 V12 V13 V14 V15 V16 V17 V18 V19 V20 V21 V22 V23 V24 V25 V26 V27 V28 V29 V30 V31 V32 V33 V34 V35 V36 V37 V38 V39 V40 V41 V42 V43) KENDALL(COMPARE=PAIRWISE)
  /MISSING SCOPE=ANALYSIS USERMISSING=EXCLUDE
  /CRITERIA ALPHA=0.05  CILEVEL=95.


Nonparametric Tests


Notes	
Output Created	07-OCT-2024 10:09:58	
Comments		
Input	Active Dataset	DataSet4	
	Filter	<none>	
	Weight	<none>	
	Split File	<none>	
	N of Rows in Working Data File	13	
Syntax	NPTESTS
  /RELATED TEST(V2 V3 V4 V5 V6 V7 V8 V9 V10 V11 V12 V13 V14 V15 V16 V17 V18 V19 V20 V21 V22 V23 V24 V25 V26 V27 V28 V29 V30 V31 V32 V33 V34 V35 V36 V37 V38 V39 V40 V41 V42 V43) KENDALL(COMPARE=PAIRWISE)
  /MISSING SCOPE=ANALYSIS USERMISSING=EXCLUDE
  /CRITERIA ALPHA=0.05  CILEVEL=95.	
Resources	Processor Time	00:00:01.06	
	Elapsed Time	00:00:01.30	


THIRD ROUND

GET DATA /TYPE=XLSX
  /FILE='C:\Users\HP\OneDrive\Desktop\DELPHI\Delphi round 3.xlsx'
  /SHEET=name 'Sheet1'
  /CELLRANGE=full
  /READNAMES=on
  /ASSUMEDSTRWIDTH=32767.
EXECUTE.
DATASET NAME DataSet5 WINDOW=FRONT.
NPAR TESTS
  /KENDALL=V2 V3 V4 V5 V6 V7 V8 V9 V10 V11 V12 V13 V14 V15 V16 V17 V18 V19 V20 V21 V22 V23 V24 V25
    V26 V27 V28 V29 V30 V31 V32 V33 V34 V35 V36 V37 V38 V39 V40 V41 V42 V43 V44 V45 V46 V47 V48 V49 V50
  /STATISTICS DESCRIPTIVES
  /MISSING LISTWISE.


NPar Tests


Notes	
Output Created	07-OCT-2024 10:18:38	
Comments		
Input	Active Dataset	DataSet5	
	Filter	<none>	
	Weight	<none>	
	Split File	<none>	
	N of Rows in Working Data File	13	
Missing Value Handling	Definition of Missing	User-defined missing values are treated as missing.	
	Cases Used	Statistics for all tests are based on cases with no missing data for any variables used.	
Syntax	NPAR TESTS
  /KENDALL=V2 V3 V4 V5 V6 V7 V8 V9 V10 V11 V12 V13 V14 V15 V16 V17 V18 V19 V20 V21 V22 V23 V24 V25
    V26 V27 V28 V29 V30 V31 V32 V33 V34 V35 V36 V37 V38 V39 V40 V41 V42 V43 V44 V45 V46 V47 V48 V49 V50
  /STATISTICS DESCRIPTIVES
  /MISSING LISTWISE.	
Resources	Processor Time	00:00:00.02	
	Elapsed Time	00:00:00.03	
	Number of Cases Alloweda	29127	

a. Based on availability of workspace memory.	


[DataSet5] 


Descriptive Statistics	
	N	Mean	Std. Deviation	Minimum	Maximum	
1	13	3.92	.277	3	4	
2	13	3.92	.277	3	4	
3	13	3.92	.277	3	4	
4	13	3.85	.376	3	4	
5	13	3.46	.519	3	4	
6	13	3.38	1.121	1	4	
7	13	3.08	1.115	1	4	
8	13	3.92	.277	3	4	
9	13	3.85	.376	3	4	
10	13	3.69	.480	3	4	
11	13	3.85	.376	3	4	
12	13	3.77	.439	3	4	
13	13	3.85	.376	3	4	
14	13	3.85	.376	3	4	
15	13	3.62	.506	3	4	
16	13	3.77	.599	2	4	
17	13	3.85	.376	3	4	
18	13	3.31	1.032	1	4	
19	13	3.38	1.044	1	4	
20	13	3.77	.439	3	4	
21	13	3.92	.277	3	4	
22	13	3.38	.961	1	4	
23	13	3.85	.376	3	4	
24	13	3.54	.776	2	4	
25	13	3.69	.480	3	4	
26	13	3.85	.376	3	4	
27	13	3.77	.439	3	4	
28	13	3.85	.376	3	4	
29	13	3.69	.480	3	4	
30	13	4.00	.000	4	4	
31	13	4.00	.000	4	4	
32	13	4.00	.000	4	4	
33	13	3.31	.480	3	4	
34	13	2.46	.660	1	3	
35	13	3.23	.725	2	4	
36	13	3.08	.641	2	4	
37	13	3.31	.480	3	4	
38	13	3.08	.760	2	4	
39	13	3.54	.519	3	4	
40	13	3.46	.967	1	4	
41	13	3.54	.519	3	4	
42	13	3.46	.660	2	4	
43	13	3.69	.480	3	4	
44	13	3.92	.277	3	4	
45	13	4.00	.000	4	4	
46	13	3.69	.480	3	4	
47	13	3.77	.439	3	4	
48	13	3.92	.277	3	4	
49	13	4.00	.000	4	4	


Kendall's W Test


Ranks	
	Mean Rank	
1	30.31	
2	30.04	
3	30.08	
4	28.19	
5	19.54	
6	23.12	
7	16.81	
8	30.15	
9	28.31	
10	25.15	
11	28.35	
12	26.58	
13	28.42	
14	28.23	
15	23.31	
16	27.73	
17	28.31	
18	20.92	
19	22.73	
20	26.58	
21	30.19	
22	21.73	
23	28.31	
24	23.73	
25	24.73	
26	28.23	
27	26.46	
28	28.23	
29	25.08	
30	31.92	
31	31.92	
32	31.92	
33	16.00	
34	5.04	
35	16.54	
36	13.92	
37	15.81	
38	13.96	
39	21.50	
40	23.54	
41	21.50	
42	20.69	
43	25.15	
44	30.19	
45	31.92	
46	24.88	
47	26.92	
48	30.19	
49	31.92	


Test Statistics	
N	13	
Kendall's Wa	.291	
Chi-Square	181.373	
df	48	
Asymp. Sig.	.000	

a. Kendall's Coefficient of Concordance	

*Nonparametric Tests: Related Samples.
NPTESTS
  /RELATED TEST(V2 V3 V4 V5 V6 V7 V8 V9 V10 V11 V12 V13 V14 V15 V16 V17 V18 V19 V20 V21 V22 V23 V24 V25 V26 V27 V28 V29 V30 V31 V32 V33 V34 V35 V36 V37 V38 V39 V40 V41 V42 V43 V44 V45 V46 V47 V48 V49 V50) KENDALL(COMPARE=PAIRWISE)
  /MISSING SCOPE=ANALYSIS USERMISSING=EXCLUDE
  /CRITERIA ALPHA=0.05  CILEVEL=95.


Nonparametric Tests


Notes	
Output Created	07-OCT-2024 10:19:20	
Comments		
Input	Active Dataset	DataSet5	
	Filter	<none>	
	Weight	<none>	
	Split File	<none>	
	N of Rows in Working Data File	13	
Syntax	NPTESTS
  /RELATED TEST(V2 V3 V4 V5 V6 V7 V8 V9 V10 V11 V12 V13 V14 V15 V16 V17 V18 V19 V20 V21 V22 V23 V24 V25 V26 V27 V28 V29 V30 V31 V32 V33 V34 V35 V36 V37 V38 V39 V40 V41 V42 V43 V44 V45 V46 V47 V48 V49 V50) KENDALL(COMPARE=PAIRWISE)
  /MISSING SCOPE=ANALYSIS USERMISSING=EXCLUDE
  /CRITERIA ALPHA=0.05  CILEVEL=95.	
Resources	Processor Time	00:00:00.78	
	Elapsed Time	00:00:00.84	
